# Assessing the Relationship between Physical Health, Mental Health and Students’ Success among Universities in Lebanon: A Cross-Sectional Study

**DOI:** 10.3390/ijerph21050597

**Published:** 2024-05-05

**Authors:** Samer A. Kharroubi, Nayla Al-Akl, Sarah-Joe Chamate, Tarek Abou Omar, Rouba Ballout

**Affiliations:** 1 Office of Student Affairs, American University of Beirut, P.O. BOX 11-0236, Riad El Solh, Beirut 1107-2020, Lebanon; na143@aub.edu.lb (N.A.-A.); sc50@aub.edu.lb (S.-J.C.); ta57@aub.edu.lb (T.A.O.); 2 Department of Nutrition and Food Sciences, Faculty of Agricultural and Food Sciences, American University of Beirut, P.O. Box 11-0236, Riad El Solh, Beirut 1107-2020, Lebanon; rb166@aub.edu.lb; 3 Department of Landscape Design and Ecosystem Management, Faculty of Agricultural and Food Sciences, American University of Beirut, P.O. Box 11-0236, Riad El Solh, Beirut 1107-2020, Lebanon

**Keywords:** university students, student success, mental health, physical health, Lebanon

## Abstract

Background: Achieving high academic success is known to be influenced by many factors including, but not limiting to, physical and mental health. The present study aimed to assess the relationship between physical health, mental health, and university students’ success, and to explore the associations between these factors and their academic achievement. Methods: A cross-sectional, self-administered online survey was used to collect data from college students in three different universities in Lebanon during the Fall 2023 semester. Mental health was evaluated using validated screening tools for depression, anxiety, and stress, specifically the Patient Health Questionnaire (PHQ-9), the General Anxiety Disorder (GAD-7), and Cohen’s Perceived Stress Scale (PSS), respectively. Additionally, general questions regarding physical health and lifestyle factors were incorporated into the questionnaire. Academic achievement was measured using students’ grade point average (GPA). Results: A total of 261 students completed the self-administered online survey. The results revealed that approximately 42% and 36% of students were experiencing moderate to severe symptoms of depression and anxiety, respectively, and 75.1% of students exhibited symptoms of moderate stress. The majority of participants (99.2%) did not report any physical disability. Chi-square analysis revealed a significant association between mental health status (depression, anxiety, and stress) and GPA level (*p* = 0.03, *p* = 0.044, *p* = 0.015, respectively). Multiple logistic regression models identified eight correlates of GPA and highlighted the relationship between physical health and student success. For instance, students who considered themselves moderately active had lower odds of achieving a higher GPA than those who considered themselves active (OR = 0.41, *p* = 0.045). Conclusions: This is the first investigation into Lebanese university students’ academic success in relation to lifestyle and mental health profiles. The findings indicate that implementing public health programs and interventions targeting mental health and lifestyle behaviors is essential for enhancing student success.

## 1. Introduction

University students play a crucial role in advancing knowledge and fostering progress in society. As future professionals, researchers, leaders, and policymakers, they hold the potential to positively impact the world [1]. Academic achievement, a concept that is essential to students’ future success, is defined as the degree to which students meet their academic objectives [2]. Unlike many similar studies, which mainly used subjective measures of academic performance, in our study, academic achievement was measured by GPA [3], which enhances the reliability of our findings. It is notable that students vary in their academic performance due to several factors, considering the numerous challenges faced by college students [4]. Addressing these factors could contribute to enhancing students’ success.

Poor mental health among college students is becoming an increasing concern for public health and policy [5,6]. The rise in mental illness, particularly among university students, emphasizes the need to comprehend risk factors and potential solutions for an environment that supports mental health. Depression, a major mental health problem, has a remarkable impact on students’ ability to perform life activities [7]. Therefore, it is necessary to explore the association between mental health and students’ productivity, academic performance, and success. Additionally, university students deal with unstable living conditions and make adaptations to changes in their environment, diet, and lifestyle [8]. A common phenomenon among college students is weight gain during their initial years. For example, Kasparek et al.’s study revealed an average weight gain of 1.3–3.1 kg among freshmen during their first term at university [9]. These unhealthy eating habits and sedentary lifestyle choices may have adverse effects on health in adulthood [10]. Moreover, many students deal with various physical illnesses, including chronic diseases and poor nutrition [11]. This emphasizes the significance of prioritizing physical well-being among university students and recognizing its association with academic success.

Lebanon, a small middle-income country in the Middle East and North Africa (MENA) region, is struggling with a significant crisis, particularly in the aftermath of the economic meltdown in 2019, the COVID-19 pandemic, and the Beirut Port explosion in 2020. It is also essential to consider Lebanon’s distinct socio-cultural background, which is distinguished by its diverse population, encompassing various cultures and religious affiliations [12]. These crises, coupled with the country’s chaotic history, have had a profound impact on the mental health and overall well-being of the population [13], as well as on the education and lifestyle of university students [14,15]. The study findings would be relevant for neighboring countries facing similar challenges. A 2022 study on a sample of university students in Lebanon revealed alarming statistics, with 22.6% and 34.4% of students exhibiting severe symptoms of depression and anxiety, respectively [16]. Another study by Fawaz et al. showed that 17.9% of Lebanese students experienced mild depression, 13.8% moderate depression, and 1.7% severe depression. Additionally, 21.9% of students reported moderate anxiety, 6.3% severe anxiety, and 2.3% extreme anxiety [17]. Among pharmacy students in Lebanon, a separate study found that 41.8% experienced severe or extremely severe anxiety, 30.7% reported depression, 28.5% faced stress, 27.7% dealt with moderate/severe insomnia, and 45.5% exhibited symptoms of PTSD [18]. 

To date, only one study has explored the association between health behaviors, mental health, and academic achievement [19]. However, academic success was measured using the Subjective Academic Achievement Scale (SAAS). The findings indicated a significant association between a higher frequency of dining out and increased psychological distress with lower SAAS scores. To our knowledge, there is a gap in research regarding the correlation between mental and physical health and GPA among college students in Lebanon, as no previous studies have examined this. Thus, the present study aims to address this research gap in Lebanon. This approach will provide a more objective assessment of students’ performance. The key objective of this study is to assess the relationship between physical health, mental health, and university students’ success, and to explore the associations between sociodemographic factors and academic achievement.

## 2. Materials and Methods

### 2.1. Study Design and Sampling

A cross-sectional study consisting of a random sampling procedure was conducted during the Fall semester of 2023 (October–December), involving students from three different universities across Lebanon. These universities included two renowned private institutions, the American University of Beirut (AUB) and the University of Balamand (UOB), as well as the Lebanese University, which is the sole public university with multiple branches and majors throughout the country. Based on a similar study conducted by Baert et al. [20], the required sample size was determined to be 220, with a 95% confidence interval and a precision level of 40%. To accommodate an additional 20% refusal rate, a total of 264 college students were included in this study. The sample size was stratified according to university size and gender, resulting in the need for a total of 137 women and 127 men for this study. 

### 2.2. Data Collection

After obtaining approval from the Dean of the Student Affairs Office at the respective universities (to avoid any undue influence or coercion), the research team visited the universities included in the study. They randomly approached students and invited them to participate in a self-administered online questionnaire. Upon agreeing to take part in the study and reviewing the consent form (Supplementary File S1), participants proceeded to complete the survey (Supplementary File S2). The survey’s completion took approximately 10 min.

Participation in the survey was entirely optional and anonymous. Additionally, participants were encouraged to ask questions related to the study or seek additional clarification before consenting to participate. Moreover, the study received approval from the Institutional Review Board (IRB) at AUB, and the research team was Collaborative Institutional Training Initiative (CITI)-certified.

### 2.3. Survey Format

The survey aimed to assess the mental and physical health of university students, along with factors associated with student success. According to the Cambridge University Reporter, examination performance is commonly used to assess academic performance [21]. In this study, academic achievement was measured by GPA, reflecting students’ performance in assignments, tests, and exams. A higher score indicates better academic performance [2]. The questionnaire was created based on similar studies in the literature [16,22] and comprised four sections. The first section consisted of questions about participants’ sociodemographic characteristics and university-related factors, including age, gender, area of residency, educational level, and major.

Section 2 included validated and reliable scales that are used to assess mental health on an ordinal scale: the Patient Health Questionnaire (PHQ-9) for depression, the General Anxiety Disorder (GAD-7) for anxiety, and Cohen’s Perceived Stress Scale (PSS) for stress. This section also encompassed some questions related to social support. The PHQ-9 comprises 9 statements on a 4-point Likert-type scale ranging from 0 (not at all) to 3 (nearly every day). For each respondent, the assigned values for PHQ-9 items were added to create a score ranging from 0 to 27. The resulting score was utilized to categorize individuals into different levels of depression: minimal (0–4), mild (5–9), moderate (10–14), moderately severe (15–19), and severe depression (20–27) [23]. The GAD-7 is a 7-item self-reported anxiety scale, where each item is graded on the Likert scale from 0 (not at all) to 3 (nearly every day). For each respondent, the assigned values for GAD-7 items were summed to create a score ranging from 0 to 21. This score was used to classify individuals into categories of minimal (0–4), mild (5–9), moderate (10–14), and severe anxiety (15–21), respectively [24]. The PSS consists of 10 items, with responses rated on the Likert scale from 0 (never) to 4 (very often). For questions 4, 5, 7, and 8, the assigned values were reversed (i.e., 0 = 4, 1 = 3, 2 = 2, 3 = 1, 4 = 0). For each respondent, the assigned values were then summed. The total scores of the PSS ranged from 0 to 40, with scores of 0-13 representing low stress, 14-26 indicating moderate stress, and 27–40 signifying high perceived stress [25].

The third section focused on the participants’ physical health (for instance, BMI) and lifestyle factors (for instance, physical activity, sleeping habits, and diet), whereas the last section included questions on students’ success. The questionnaire was pilot tested on 10 students to check for clarity. Data collected during the pilot testing phase were not incorporated into the present study. Supplementary File S2 contains a copy of the questionnaire used for data collection.

### 2.4. Statistical Analysis

Data were rigorously checked for completeness and were then entered into the Statis-tical Package for the Social Sciences (SPSS) version 29.0 (SPSS Inc., Chicago, IL, USA) for data analysis. Descriptive statistics were used, such as counts and percentages, for the categorical variables and means and standard deviations (SD) for the continuous ones. Chi-square (χ2) was used to calculate the association between two categorical variables. Note that GPA for UOB students was multiplied by 0.98, whereas for LU students, it was multiplied by 1.2 in accordance with AUB equivalence system. GPA was then dichotomized. The mean GPA score was considered the cut-point. Participants reporting a GPA below the mean score, i.e., between 0 and 79, were considered to have a low to moderate GPA, and those reporting a GPA above or equal to the mean score, i.e., 80 and above, were considered to have a high GPA.

Simple and multiple logistic regression were applied to investigate which factors were associated with student success, using the GPA score as an dependent variable and the sociodemographic factors, such as mental and physical health and university, as independent variables. All variables that were found to be significant in the simple analysis were added simultaneously to the multiple regression models as independent variables. Results from the logistic regression analyses were expressed as odds ratios (ORs) with their respective 95% confidence intervals (CIs). A *p*-value below 0.05 was considered statistically significant in all analyses.

## 3. Results

### 3.1. Sociodemographic Characteristics 

A total of 264 students completed the online survey, with 261 providing complete data and being included in the final analysis (resulting in a 99% completion rate). The sociodemographic characteristics of the study population, along with some information about their studies, are presented in Table 1. Based on the gender and university stratification plan, approximately 52% of the students were women, with 27.6% enrolled in AUB, 51.3% in LU, and 21.1% in UOB.

The average age of the college students was 20.29 years (SD = 2.025), ranging between 17 and 33 years old. The majority of students were from Beirut (40.2%) and Mount Lebanon (31%). A significant portion of the participants were Lebanese (92.3%), and many were enrolled in non-health-related majors (73.6%). About 64.4% of the participants lived with their families. Additionally, around 36% of the students reported a personal monthly income between USD 100 and 300. Nearly half of the participants’ parents held a university degree. Most students had working fathers (92.3%), while 56.7% had working mothers. Moreover, more than two-thirds of the participants had no employment (72.4%) and did not receive any financial aid or scholarship (67%). Approximately 32% reported studying more than 30 hours per week. Finally, 48.7% of college students had a low to moderate GPA, while 51.3% had a high GPA.

### 3.2. Mental Health of Participants and Its Association with GPA

Among the study sample (Table 2), 73.5% of students exhibited mild to moderately severe symptoms of depression, and 80.5% displayed mild to severe symptoms of anxiety. Specifically, 44.1% showed mild symptoms of anxiety, while 10.7% exhibited severe symptoms. Approximately 75.1% presented with moderate symptoms of stress, and 13.4% of students exhibited high perceived stress. More than half of the participants (64.7%) believed that their mental health had a negative impact on their ability to learn, focus, and perform well in university.

As indicated in Table 2, significant differences were observed in the mental health status (depression, anxiety, and stress) of college students based on their GPA level. The rating of the negative impact of mental health was also significantly associated with GPA level.

### 3.3. Physical Health of Participants and Its Association with GPA

As shown in Table 3, the majority of college students in the study sample did not report any physical disability (99.2%) or chronic disease (83.1%). Among those who reported chronic disease, asthma was the most prevalent (38.6%). About 52.1% of participants experienced frequent headaches, fatigue, or stomachaches. Only 36.4% considered themselves physically active, while 45.6% reported being moderately active, and 18% described themselves as sedentary. Moreover, 47.9% of students went to the gym, 38.3% were daily smokers, and 33.3% consumed alcohol, with 24.5% doing so less than two times per week. Approximately 14.2% of participants identified as underweight, while 11.5% considered themselves obese. More than half of the participants (59.4%) were satisfied with their weight, and 52.5% reported weight fluctuations during their university years. Additionally, 65.9% of students reported having regular sleeping habits, and 57.5% believed they followed healthy eating habits, with homemade food being the most preferred option (42.5%). Only 32.9% of participants believed that their physical health negatively impacted their ability to learn, focus, and perform well in university. Finally, GPA was significantly associated with physical activity, smoking habits, gym attendance, and weight satisfaction.

### 3.4. University Factors and Their Association with GPA

As shown in Table 4, a significant portion of the participants (79.3%) never thought of dropping out of university. More than half of them (59.4%) believed they would complete their studies on time. Approximately 40% engaged in internships or other opportunities to gain practical experience in their fields and actively participated in extracurricular activities (ECAs). Furthermore, 66.7% of students expressed satisfaction with the quality of education and the learning environment provided by their institution, while 54.8% were pleased with the quality of services offered by the student affairs office at their institution. Finally, the results revealed a significant association between GPA and the likelihood of finishing studies on time, as well as satisfaction with the quality of education provided by the institution.

### 3.5. Simple and Multiple Logistic Regression Analyses

Simple logistic regression analysis revealed that fifteen predictors were significantly associated with participants’ GPA level (Table 5). These predictors included the area of residency (OR = 0.396, *p* = 0.002), where participants from Mount Lebanon were less likely to have a better GPA than those residing in Beirut. Other significant predictors comprised the stage of study (OR = 2.718, *p* = 0.036), major (OR = 1.836, *p* = 0.035), mother’s employment status (OR = 3.904, *p* = 0.01), university enrollment (OR = 0.377, *p* = 0.009), PHQ9 (mild depression: OR = 0.448, *p* = 0.025; moderately severe depression: OR = 0.26, *p* = 0.002), GAD7 (OR = 0.338, *p* = 0.005), PSS (OR = 0.35, *p* = 0.045), negative impact of mental health (a great deal: OR = 2.271, *p* = 0.01; some: OR = 2.667, *p* = 0.004), physical activity (moderately active: OR = 0.501, *p* = 0.014; sedentary: OR = 0.396, *p* = 0.011), gym (OR = 0.479, *p* = 0.004), smoking (OR = 1.968, *p* = 0.009), weight satisfaction (OR = 0.584, *p* = 0.034), finishing study on time (OR = 0.47, *p* = 0.024), and satisfaction with the quality of education provided by the university (OR = 0.547, *p* = 0.023).

The results from the multiple logistic analysis revealed several significant associations with participants’ GPA levels. More specifically, participants with a health-related major were more likely to have a high GPA compared to those with a non-health major (OR = 3.874, *p* = 0.007). Students studying at UOB had lower odds of having a high GPA compared to those studying at AUB (OR = 0.222, *p* = 0.004). Additionally, participants who reported being moderately active were less likely to have a high GPA compared to those who reported being active (OR = 0.41, *p* = 0.045). Students who do not smoke daily were more likely to have a high GPA compared to those who smoke daily (OR = 2.948, *p* = 0.003). Further, participants who reported being unsatisfied with the quality of education and the learning environment provided by their institution were less likely to have a high GPA compared to those who reported being satisfied (OR = 0.439, *p* = 0.033). Also, students who have working mothers specifically self-employed were more likely to have a high GPA compared to those who have non-working mothers (OR = 3.971, *p* = 0.043). Finally, the results showed that the student’s area of residency, and whether they consider that mental health has a negative impact on their studies were all significantly associated with their GPA level (OR = 0.363, *p* = 0.011; OR = 3.019, *p* = 0.038, respectively).

## 4. Discussion

### 4.1. General Findings

The present study is among the few conducted in the MENA region, including Lebanon, that investigates the correlations between mental health, physical health, and academic achievement. To the best of our knowledge, this is the first study in Lebanon to employ GPA as a measure of students’ success, departing from more subjective methods such as the Subjective Academic Achievement Scale (SAAS) [19].

Our results revealed alarming rates of depression and anxiety, surpassing those documented in previous studies. Specifically, our findings indicated that nearly 76% of students exhibited mild to moderately severe symptoms of depression, and approximately 70% reported mild to moderate symptoms of anxiety. In contrast, studies conducted in 2018 showed that 56% of students were experiencing mild to moderate depression symptoms, with 36% and 34% presenting combined symptoms of depression and anxiety, respectively [26,27]. Despite using identical mental health indicators, these variations may be attributed to methodological differences, particularly the focus on single universities in the latter studies. The ongoing crisis in the country could be another influencing factor contributing to the increased prevalence of mental health issues among young adults, especially since the previous studies were conducted before the economic crisis and the onset of the COVID-19 pandemic. Furthermore, the levels of anxiety reported in our study were higher than those documented among college students in Lebanon in 2021 (50%) [16], the United States (15.9%) [26], and Canada (32.6%) [28]. Additionally, approximately 75% of our study sample exhibited moderate stress levels, a figure higher than the reported levels among pharmacy students in Lebanon (27.7%) [18].

The present study also revealed that a significant percentage of students reported a negative impact of their mental health on academic performance. This finding is consistent with a Student Voice survey on health and wellness conducted by Inside Higher Ed and College Pulse, where half of the students asserted that their well-being adversely affects their academic progress [29]. In a longitudinal study conducted in the United States, it was found that mental health problems predicted delayed academic success (GPA) [30]. It is worth noting that mental health issues and their relationship with students’ success have not received sufficient attention in the MENA region compared to other countries [31]. The present study’s findings highlight a significant association between mental health and student success. These findings highlight the urgent need for comprehensive mental health support services on university campuses to address the well-being of students and mitigate the adverse effects on their academic success.

Our findings indicate that only 11.5% of students perceive themselves as overweight or obese, a considerably lower proportion compared to other studies. For example, a cross-sectional study encompassing university students from 22 low-, middle-income, and emerging economy countries reported that 22% of participants were overweight or obese [32]. Similarly, a study conducted in Egypt found that approximately one quarter of males and one third of females fell into the overweight/obese category [31]. A potential explanation for our results may be attributed to self-reported bias, as students were required to assess their weight based on BMI. Additionally, the mean BMI of college students in our study sample was 22.41 ± 3.37, aligning with acceptable ranges observed in previous studies conducted in Lebanon, Morocco, and Saudi Arabia [19,33,34,35]. Although our study did not identify any association between BMI and GPA, it is noteworthy that several studies have indicated a negative relationship between BMI and academic achievement [36,37]. Future studies should examine the complex relationship between academic achievement and weight status, accounting for variables like health behaviors and body image perception.

Our results indicated an association between GPA and the area of residency, contrary to findings in other studies. For instance, Alfifi et al. discovered that the residential area has no significant influence on academic achievement [38,39,40]. In contrast, their research found that women outperformed male students, while our findings revealed no gender-based differences in GPA among college students in the study sample. Additionally, in line with previous studies, our results demonstrated that students pursuing health-related majors were more likely to achieve higher GPAs compared to non-health majors [39,40]. This differs from a study conducted in Lebanon, where better academic achievement was observed among students with non-scientific majors, potentially influenced by the subjective method used to measure academic achievement in that region [19]. It follows from previous research that parents’ socio-economic status, including academic and professional qualifications and income, can be associated with student success [22]. However, in the present study, only the mother’s employment status was found to be significantly associated with GPA. The university in which students were enrolled was also identified as a factor affecting GPA, possibly attributed to varying resources and facilities offered by each institution. This finding aligns with the significant association observed between students’ satisfaction with the quality of education provided by their institution and GPA. Regarding physical and lifestyle factors, a significant association was revealed between smoking and GPA, consistent with lower GPAs among students who smoke, as reported in previous studies [19,41]. Physical activity also showed an association with GPA, with sedentary students being less likely to achieve high GPAs compared to their active counterparts. This association was previously highlighted in a study among medical students in Saudi Arabia [42].

Sleep deprivation is common among university students and has been linked to poor academic performance [43]. While a study in Belgium found a positive relationship between sleep quality and academic achievement [20], no association was found between sleeping habits or hours and GPA in our study.

### 4.2. Scientfic and Practical Recommendations

The results of our study have consequences not just for Lebanon but also for other nearby countries in the MENA region, where problems with university students’ mental, physical, and academic well-being may be common. The MENA region shares certain issues including economic instability, political turmoil, and the effects of global health crises [44]. Thus, the knowledge gathered from our research could guide the development of interventions and policies meant to promote the academic achievement and general well-being of university students in surrounding countries.

Moreover, our research highlights the necessity of paying more attention to mental health concerns among college students worldwide. The concerning rates of stress, anxiety, and depression that our research revealed are likely not specific to Lebanon; rather, they could be a reflection of larger patterns that influence young adults across a range of cultural contexts [45]. Because of this, international efforts to address mental health on college campuses should be prioritized, with an emphasis on developing supportive learning environments, facilitating access to mental health services.

### 4.3. Research Limitations

The findings of the current study should be interpreted considering the study’s limitations. First, our study was cross-sectional; causal relationships cannot be drawn. For instance, students from low-income families may face unique challenges such as limited access to opportunities for physical activities, which could affect their academic performance. Moreover, time constraints may be associated with students’ ability to dedicate sufficient time to learning. Therefore, future studies using longitudinal designs could be developed to better understand causal relationships. Second, the data collection process utilized self-reported responses to assess the relationship between both physical and mental health factors and the academic success of university students. However, it is important to acknowledge the potential for inaccuracies stemming from memory recall or social desirability bias within these responses. Third, this study primarily employed quantitative methods for assessment. Future research endeavors may benefit from incorporating qualitative approaches to further explore the relationship between physical health, mental health, and university students’ success, thereby offering a more comprehensive understanding of the subject matter.

## 5. Conclusions

The present study shed light on the alarming levels of depression, anxiety, and stress reported among college students. Our findings highlighted the association between mental and physical health with GPA, emphasizing the critical need for targeted interventions to support student well-being and academic success. Policymakers and universities must develop and implement awareness campaigns and health education initiatives tailored specifically to the needs of university students. In order to reduce the negative impact of mental health issues on academic performance, these programs should emphasize the promotion of a healthy lifestyle, which includes regular physical activity, a balanced diet, and enough sleep. Furthermore, it is critical to create a welcoming campus environment that promotes open dialogue about mental health and easily accessible options for students in need of support. 

## Figures and Tables

**Table 1:** Sociodemographic characteristics and university factors of college students in Lebanon in the study sample (*n* = 261).

Characteristics		*n* (%)	Low to Moderate GPA *n* (%)	High GPA *n* (%)	*p*-Value
Gender ^a^	Man	126 (48.3)	63 (50)	63 (50)	0.675
	Women	135 (51.7)	64 (47.4)	71 (52.6)	
Age ^a^	Mean: 20.3	SD: 2			
Area of residency ^a^	Beirut	105 (40.2)	45 (52.9)	60 (57.1)	**0.006**
	Mount Lebanon	81 (31)	53 (65.4)	28 (34.6)	
	South	29 (11.1)	13 (44.8)	16 (55.2)	
	North	28 (10.7)	10 (35.7)	18 (64.3)	
	Bekaa	18 (6.9)	6 (33.3)	2 (66.7)	
Nationality ^a^	Lebanese	241 (92.3)	114 (47.3)	127 (52.7)	0.128
	Non-Lebanese	20 (7.7)	13 (65)	7 (35)	
Stage of study ^b^	1st semester	48 (18.4)	26 (54.2)	22 (45.8)	0.258
	2nd–3rd semester	65 (24.9)	33 (50.8)	32 (49.2)	
	4th–5th semester	59 (22.6)	30 (50.8)	29 (49.2)	
	6th–8th semester	56 (21.5)	28 (50)	28 (50)	
	9th + semester	33 (12.6)	10 (30.3)	23 (69.7)	
Major ^b^	Non-health-related	192 (73.6)	101 (52.6)	91 (47.4)	**0.033**
	Health-related	69 (26.4)	26 (37.7)	43 (62.3)	
Where do you live during university years? ^a^	Family	168 (64.4)	86 (51.2)	82 (48.8)	0.542
	Roommates	72 (27.5)	32 (44.4)	40 (55.6)	
	Alone	21 (8)	9 (42.9)	12 (57.1)	
Personal monthly income/allowance (USD) ^a^	<100	82 (31.4)	42 (51.2)	40 (48.8)	0.137
	100–300	93 (35.6)	45 (48.4)	48 (51.6)	
	300–500	49 (18.8)	28 (57.1)	21 (42.9)	
	≥500	37 (14.2)	12 (32.4)	25 (67.6)	
Monthly income of household (USD) ^a^	<500	38 (14.6)	17 (44.7)	21 (55.3)	0.822
	500–800	50 (19.2)	27 (54)	23 (46)	
	800–1000	69 (26.4)	34 (49.3)	35 (50.7)	
	≥1000	104 (39.8)	49 (47.1)	55 (52.9)	
Education of father ^a^	Intermediate or less	41 (15.7)	18 (43.9)	23 (56.1)	0.824
	High school	66 (25.3)	35 (53)	31 (47)	
	University	137 (52.5)	66 (48.2)	71 (51.8)	
	Other	17 (6.5)	8 (47.1)	9 (52.9)	
Education of mother ^a^	Intermediate or less	26 (9.9)	14 (53.8)	12 (46.2)	0.719
	High school	66 (25.3)	35 (53)	31 (47)	
	University	149 (57.1)	68 (45.6)	81 (54.4)	
	Other	20 (7.7)	10 (50)	10 (50)	
Father’s employment status ^a^	Not working	20 (7.7)	8 (40)	12 (60)	0.703
	Self-employed	97 (37.2)	47 (48.5)	50 (51.5)	
	Employed	144 (55.1)	72 (50)	72 (50)	
Mother’s employment status ^a^	Not working	148 (56.7)	77 (52)	71 (48)	**0.025**
	Self-employed	23 (8.8)	5 (21.7)	18 (78.3)	
	Employed	90 (38.5)	45 (50)	45 (50)	
Your current job ^a^	No job	189 (72.4)	87 (46)	102 (54)	0.169
	Employed	72 (27.6)	40 (55.6)	32 (44.4)	
Time spent on study (per week) ^b^	<10 h	34 (13)	14 (41.2)	20 (58.8)	0.217
	10–19 h	70 (26.8)	41 (58.6)	29 (41.4)	
	20–29 h	74 (28.4)	36 (48.6)	38 (51.4)	
	30+ h	83 (31.8)	36 (43.4)	47 (56.6)	
Receive financial aid or scholarship? ^b^	Yes	86 (33)	41 (47.7)	45 (52.3)	0.823
	No	175 (67)	86 (49.1)	89 (50.9)	
Do you agree that your life is stressful? ^a^	1 (Strongly disagree)	7 (2.7)	3 (42.9)	4 (57.1)	0.094
	2	33 (12.6)	15 (45.5)	18 (54.5)	
	3	72 (27.6)	26 (36.1)	46 (63.9)	
	4	85 (32.6)	46 (54.1)	39 (45.9)	
	5 (Strongly agree)	64 (24.5)	37 (57.8)	27 (42.2)	
University enrolled in ^b^	American University of Beirut	72 (27.6)	30 (41.7)	42 (58.3)	**0.017**
	Lebanese University	134 (51.3)	61 (45.5)	73 (54.5)	
	University of Balamand	55 (21.1)	36 (65.5)	19 (34.5)	
GPA ^1,b^	Low to moderate	127 (48.7)			
	High	134 (51.3)			
BMI ^2,a^	Mean: 22.4	SD: 3.4			

^a^ Sociodemographic characteristics, ^b^ university factors, ^1^ GPA: grade point average; ^2^ BMI: body mass index. Estimates shown in bold are those that are statistically significant at *p* < 0.05.

**Table 2:** Mental health of college students (*n* = 261).

Characteristics		*n* (%)	Low to Moderate GPA *n* (%)	High GPA *n* (%)	*p*-Value
Patient Health Questionnaire (PHQ9)	Minimal depression (1–4)	52 (19.9)	17 (32.7)	35 (67.3)	**0.03**
	Mild depression (5–9)	98 (37.5)	51 (52)	47 (48)	
	Moderate depression (10–14)	51 (19.5)	23 (45.1)	28 (54.9)	
	Moderately severe depression (15–19)	43 (16.5)	28 (65.1)	15 (34.9)	
	Severe depression (20+)	17 (6.5)	8 (47.1)	9 (52.9)	
General Anxiety Disorder (GAD7)	Minimal anxiety (0–4)	51 (19.5)	17 (33.3)	34 (66.7)	**0.044**
	Mild anxiety (5–9)	115 (44.1)	56 (48.7)	59 (51.3)	
	Moderate anxiety (10–14)	67 (25.7)	40 (59.7)	27 (40.3)	
	Severe anxiety (15+)	28 (10.7)	14 (50)	14 (50)	
Cohen’s Perceived Stress Scale (PSS)	Low stress (0–13)	30 (11.5)	14 (46.7)	16 (53.3)	**0.015**
	Moderate stress (14–26)	196 (75.1)	88 (44.9)	108 (55.1)	
	High perceived stress (27–40)	35 (13.4)	25 (71.4)	10 (28.6)	
Number of people that support you when you feel down	0	14 (5.4)	6 (42.9)	8 (57.1)	0.089
	1	87 (33.3)	52 (59.8)	35 (40.2)	
	2–3	97 (37.2)	41 (42.3)	56 (57.7)	
	>3	63 (24.1)	28 (44.4)	35 (55.6)	
Satisfaction with this support	1 (very unsatisfied)	15 (5.7)	9 (60)	6 (40)	0.126
	2	23 (8.8)	16 (69.6)	7 (30.4)	
	3	67 (25.7)	35 (52.2)	32 (47.8)	
	4	76 (29.1)	33 (43.4)	43 (56.6)	
	5 (very satisfied)	80 (30.7)	34 (42.5)	46 (57.5)	
Negative impact of mental health on your ability to focus, learn and do well in university	A great deal	76 (29.1)	48 (63.2)	28 (36.8)	**0.035**
	Some	93 (35.6)	40 (43)	53 (57)	
	Not too much	69 (26.4)	27 (39.1)	42 (60.9)	
	Not at all	18 (6.9)	9 (50)	9 (50)	
	Not sure	5 (1.9)	3 (60)	2 (40)	

Estimates shown in bold are those that are statistically significant at *p* < 0.05.

**Table 3:** Physical health of college students (*n* = 261).

Characteristics		*n* (%)	Low to Moderate GPA *n* (%)	High GPA *n* (%)	*p*-Value
Do you suffer from any physical disability?	Yes	2 (0.8)	2 (100)	0 (0)	0.145
	No	259 (99.2)	125 (48.3)	134 (51.7)	
Do you suffer from any chronic disease?	Yes	44 (16.9)	23 (52.3)	21 (47.7)	0.599
	No	217 (83.1)	104 (47.9)	113 (52.1)	
Which chronic disease do you suffer from? (*n* = 44)	Asthma	17 (38.6)	-	-	
	Obesity	3 (6.8)			
	Hypertension	4 (9)			
	Poor oral health	1 (2.3)			
	Digestive system diseases	7 (15.9)			
	Diabetes	5 (11.4)			
	Muscle pains	2 (4.5)			
	Other	5 (11.4)			
Do you suffer frequently from headaches, stomachaches, fatigue?	Yes	136 (52.1)	71 (52.2)	65 (47.8)	0.232
	No	125 (47.9)	56 (44.8)	69 (55.2)	
Physical activity	Active	95 (36.4)	35 (36.8)	60 (63.2)	**0.012**
	Moderately active	119 (45.6)	64 (53.8)	55 (46.2)	
	Sedentary	47 (18)	28 (59.6)	19 (40.4)	
Do you go to the gym?	Yes	125 (47.9)	49 (39.2)	76 (60.8)	**0.003**
	No	136 (52.1)	78 (57.4)	58 (42.6)	
Are you a daily smoker?	Yes	100 (38.3)	59 (59)	41 (41)	**0.008**
	No	161 (61.7)	68 (42.2)	93 (57.8)	
What do you smoke? (*n* = 100)	Cigarettes	49 (49)	-	-	
	Water-pipe	27 (27)			
	Vape	19 (19)			
	All the above	4 (4)			
	Other	1(1)			
Alcohol use	<2 times per week	64 (24.5)	29 (45.3)	35 (54.7)	0.924
	2 times per week	13 (5)	7 (53.8)	6 (46.2)	
	>2 times per week	10 (3.8)	5 (50)	5 (50)	
	I don’t drink alcohol	174 (66.7)	86 (49.4)	88 (50.6)	
How do you describe your weight according to BMI?	Under weight	37 (14.2)	20 (54.1)	17 (45.9)	0.449
	Normal	194 (74.3)	90 (46.4)	104 (53.6)	
	Overweight/ Obese	30 (11.5)	17 (56.7)	13 (43.3)	
Are you satisfied with your weight?	Yes	155 (59.4)	67 (43.2)	88 (56.8)	**0.034**
	No	106 (40.6)	60 (56.6)	46 (43.4)	
Did you lose/gain any weight during your university years?	Lose	68 (26.1)	36 (52.9)	32 (47.1)	0.704
	Gain	69 (26.4)	32 (46.4)	37 (53.6)	
	No	124 (47.5)	59 (47.6)	65 (52.4)	
Daily sleep hours	≤6	65 (24.9)	30 (46.2)	35 (53.8)	0.418
	7	61 (23.4)	28 (45.9)	33 (54.1)	
	8	66 (25.3)	38 (57.6)	28 (42.4)	
	>8	69(26.4)	31 (44.9)	38 (55.1)	
Sleeping habit	Regular	172 (65.9)	83 (48.3)	89 (51.7)	0.856
	Irregular	89 (34.1)	44 (49.4)	45 (50.6)	
Do you consider yourself to follow healthy eating habits?	Yes	150 (57.5)	70 (46.7)	80 (53.3)	0.454
	No	111 (42.5)	57 (51.4)	54 (48.6)	
Which option best describes your diet?	Mostly fast/processed food	68 (26.1)	37 (54.4)	31 (45.6)	0.22
	Mostly homemade food	111 (42.5)	51 (45.9)	60 (54.1)	
	Mostly vegan or vegetarian	6 (2.3)	5 (83.3)	1 (16.7)	
	All the above	70 (26.8)	30 (42.9)	40 (57.1)	
	Other	6 (2.3)	4 (66.7)	2 (33.3)	
How often do you engage in recreational physical activity?	Never	27 (10.3)	14 (51.9)	13 (48.1)	0.561
	Rarely	147 (56.3)	76 (51.7)	71 (48.2)	
	1–2 times per week	62 (23.8)	27 (43.5)	35 (56.5)	
	3+ times per week	25 (9.6)	10 (40)	15 (60)	
How often are you in contact with nature?	Never	20 (7.7)	10 (50)	10(50)	0.462
	Rarely	177 (67.8)	91 (51.4)	86 (48.6)	
	1–2 times per week	57 (21.8)	24 (42.1)	33 (57.9)	
	3+ times per week	7 (2.7)	2 (28.6)	5 (71.4)	
Negative impact of physical health on your ability to focus, learn and do well in university	A great deal	27 (10.3)	12 (44.4)	15 (55.6)	0.41
	Some	59 (22.6)	33 (55.9)	26 (44.1)	
	Not too much	92 (35.2)	45 (48.9)	47 (51.1)	
	Not at all	74 (28.4)	31 (41.9)	43 (58.1)	
	Not sure	9 (3.4)	6 (66.7)	3 (33.3)	

Estimates shown in bold are those that are statistically significant at *p* < 0.05.

**Table 4:** University factors (*n* = 261).

Characteristics		*n* (%)	Low to moderate GPA *n* (%)	High GPA *n* (%)	*p*-Value
Have you ever thought of dropping from university?	Yes	54 (20.7)	30 (55.6)	24 (44.4)	0.255
	No	207 (79.3)	97 (46.9)	110 (53.1)	
Will you finish your study on time?	Yes	155 (59.4)	66 (42.6)	89 (57.4)	**0.046**
	No	57 (21.8)	31 (54.4)	26 (45.6)	
	Not sure	49 (18.8)	30 (61.2)	19 (38.8)	
Are you satisfied with the quality of education and the learning environment provided by your institution?	Yes	174 (66.7)	76 (43.7)	98 (56.3)	**0.023**
	No	87 (33.3)	51 (58.6)	36 (41.4)	
Are you satisfied with the quality of services provided by your student affairs at your institution?	Yes	143 (54.8)	71 (49.7)	72 (50.3)	0.724
	No	118 (45.2)	56 (47.5)	62 (52.5)	
Have you engaged in internships, or other opportunities to gain practical experience in your field?	Yes	112 (42.9)	47 (42)	65 (58)	0.061
	No	149 (57.1)	80 (53.7)	69 (46.3)	
Are you actively involved in extracurricular activities?	Yes	95 (36.4)	40 (42.1)	55 (57.9)	0.109
	No	166 (63.6)	87 (52.4)	79 (47.6)	

Estimates shown in bold are those that are statistically significant at *p* < 0.05.

**Table 5:** Simple and multiple logistic regression.

	GPA	
	Simple OR, (95% CI), *p*-Value	Multiple OR, (95% CI), *p*-Value
**Age**	1 (0.887, 1.128), 0.999	
**Gender**		
Male	1	
Female	1.109 (0.682, 1.803), 0.675	
**Nationality**		
Lebanese	1	
Non-Lebanese	0.483 (0.186, 1.254), 0.135	
**Area of residency**		
Beirut	1	1
Mount Lebanon	**0.396 (0.218, 0.721), 0.002**	**0.363 (0.166, 0.791), 0.011**
South	0.923 (0.403, 2.112), 0.850	0.912 (0.309, 2.691), 0.867
North	1.35 (0.569, 3.204), 0.496	1.201 (0.374, 3.859), 0.758
Bekaa	1.5 (0.523, 4.301), 0.451	0.603 (0.147, 2.485), 0.484
**Stage of study**		
1st semester	1	1
2nd–3rd semester	1.146 (0.543, 2.42), 0.721	1.108 (0.397, 3.095), 0.845
4th–5th semester	1.142 (0.532, 2.451), 0.732	1.225 (0.448, 3.353), 0.693
6th–8th semester	1.182 (0.546, 2.559), 0.672	1.071 (0.377, 3.041), 0.897
9th + semester	**2.718 (1.068, 6.921), 0.036**	1.558 (0.432, 5.614), 0.498
**Major**		
Non-health-related	1	1
Health-related	**1.836 (1.045, 3.224), 0.035**	**3.874 (1.455, 10.321), 0.007**
**Where do you live during your university years?**		
Family	1	
Roommates	1.311 (0.753, 2.283), 0.339	
Alone	1.398 (0.56, 3.494), 0.473	
**Personal monthly income/allowance (USD)**		
<100	1	
100–300	1.12 (0.618, 2.029), 0.708	
300–500	0.788 (0.386, 1.606), 0.511	
≥500	2.187 (0.97, 4.933), 0.059	
**Monthly income of household**		
<500	1	
500–800	0.69 (0.295, 1.609), 0.39	
800–1000	0.833 (0.376, 1.845), 0.653	
≥1000	0.909 (0.431, 1.917), 0.801	
**Education of father**		
Intermediate or less	1	
High school	0.693 (0.317, 1.518), 0.359	
University	0.842 (0.417, 1.699), 0.631	
Other	0.88 (0.283, 2.738), 0.826	
**Education of mother**		
Intermediate or less	1	
High school	1.033 (0.416, 2.567), 0.944	
University	1.39 (0.603, 3.205), 0.44	
Other	1.167 (0.363, 3.749), 0.796	
**Father’s employment status**		
Not working	1	
Self-employed	0.709 (0.266, 1.888), 0.492	
Employed	0.667 (0.257, 1.728), 0.404	
**Mother’s employment status**		
Not working	1	1
Self-employed	**3.904 (1.377, 11.068), 0.01**	**3.971 (1.043, 15.116), 0.043**
Employed	1.085 (0.642, 1.832), 0.762	1.315 (0.641, 2.698), 0.455
**Your current job**		
No job	1	
Employed	0.682 (0.395, 1.178), 0.17	
**Time spent on study**		
<10 h	1	
10 h–19 h	0.495 (0.215, 1.138), 0.098	
20 h–29 h	0.739 (0.325, 1.68), 0.47	
30+ h	0.914 (0.47, 2.053), 0.827	
**Receive financial aid or scholarship**		
Yes	1	
No	0.943 (0.562, 1.581), 0.823	
**Do you agree that your life is stressful?**		
1 (Strongly disagree)	1	
2	0.9 (0.173, 4.669), 0.9	
3	1.327 (0.275, 6.393), 0.724	
4	0.636 (0.134, 3.016), 0.636	
5 (Strongly agree)	0.547 (0.113, 2.649), 0.454	
**University enrolled in**		
AUB	1	1
Lebanese University	0.855 (0.479, 1.525), 0.595	1.81 (0.752, 4.356), 0.186
Balamand	**0.377 (0.182, 0.78), 0.009**	**0.222 (0.080, 0.617), 0.004**
**PHQ9**		
Minimal depression (1–4)	1	1
Mild depression (5–9)	**0.448 (0.222, 0.903), 0.025**	0.652 (0.187, 2.278), 0.503
Moderate (10–14)	0.591 (0.266, 1,316), 0.198	1.891 (0.41, 8.714), 0.414
Moderately severe (15–19)	**0.26 (0.111, 0.611), 0.002**	1.457 (0.263, 8.064), 0.666
Severe depression (20+)	0.546 (0.179, 1.666), 0.288	3.119 (0.28, 34.785), 0.355
**GAD7**		
Minimal anxiety (0–4)	1	1
Mild anxiety (5–9)	0.527 (0.265, 1.048), 0.068	0.486 (0.128, 1.845), 0.289
Moderate (10–14)	**0.338 (0.158, 0.722), 0.005**	0.344 (0.068, 1.733), 0.196
Severe anxiety (15+)	0.5 (0.195, 1.283), 0.149	0.58 (0.06, 5.59), 0.637
**PSS**		
Low stress (0–13)	1	1
Moderate stress (14–26)	1.074 (0.497, 2.321), 0.856	1.215 (0.295, 5), 0.788
High perceived stress (27–40)	**0.35 (0.125, 0.976), 0.045**	0.464 (0.072, 2.99), 0.42
**Number of people that support you when you feel down**		
0	1	
1	0.505 (0.161, 1.582), 0.241	
2–3	1.024 (0.33, 3.179), 0.967	
>3	0.938 (0.291, 3.019), 0.914	
**Satisfaction with this support**		
1 (very unsatisfied)	1	
2	0.656 (0.168, 2.563), 0.545	
3	1.371 (0.439, 4.283), 0.587	
4	1.955 (0.633, 6.04), 0.244	
5 (very satisfied)	2.029 (0.659, 6.245), 0.217	
**Negative impact of mental health on your ability to focus, learn and do well in university**		
A great deal	1	1
Some	**2.271 (1.221, 4.227), 0.01**	**3.019 (1.063, 8.573), 0.038**
Not too much	**2.667 (1.362, 5.219), 0.004**	2.688 (0.83, 8.702), 0.099
Not at all	1.714 (0.609, 4.825), 0.307	0.645 (0.087, 4.775), 0.668
Not sure	1.143 (0.18, 7.26), 0.887	0.227 (0.019, 2.7), 0.24
**Do you suffer from any physical disability?**		
Yes	1	
No	-	
**Do you suffer from any chronic disease?**		
Yes	1	
No	1.19 (0.622, 2.277), 0.599	
**Do you suffer frequently from headaches, stomachaches, fatigue?**		
Yes	1	
No	1.346 (0.827, 2.191), 0.232	
**Physical activity**		
Active	1	1
Moderately active	**0.501 (0.289, 0.87), 0.014**	**0.41 (0.171, 0.98), 0.045**
Sedentary	**0.396 (0.193, 0.81), 0.011**	0.594 (0.184, 1.915), 0.383
**Do you go to the gym?**		
Yes	1	1
No	**0.479 (0.292, 0.786), 0.004**	0.695 (0.321, 1.502), 0.355
**Are you a daily smoker?**		
Yes	1	1
No	**1.968 (1.186, 3.266), 0.009**	**2.948 (1.457, 5.961), 0.003**
**Alcohol use**		
<2 times per week	1	
2 times per week	0.71 (0.215, 2,349), 0.575	
>2 times per week	0.829 (0.218, 3.145), 0.782	
I don’t drink alcohol	0.848 (0.477, 1.507), 0.574	
**How do you describe your weight?**		
Under weight	1	
Normal	1.359 (0.671, 2.753), 0.394	
Overweight/Obese	0.9 (0.341, 2.372), 0.831	
**Are you satisfied with your weight?**		
Yes	1	1
No	**0.584 (0.355, 0.961), 0.034**	0.734 (0.379, 1.42), 0.358
**Did you lose/gain any weight during your university years?**		
Lose	1	
Gain	1.301 (0.665, 2.545), 0.443	
No	1.239 (0.685, 2.241), 0.478	
**Sleeping hours**		
≤6	1	
7	(0.501, 2.036), 0.977	
8	0.632 (0.317, 1.259), 0.192	
>8	1.051 (0.532, 2.075), 0.887	
**Sleeping habits**		
Regular	1	
Irregular	0.954 (0.572, 1.591), 0.856	
**Do you consider yourself to follow healthy eating habits?**		
Yes	1	
No	0.829 (0.507, 1.355), 0.454	
**Which option best describes your diet?**		
Mostly fast/processed food	1	
Mostly homemade food	1.404 (0.766, 2.574),0.272	
Mostly vegan or vegetarian	0.239 (0.026, 2.153), 0.202	
All the above	1.591 (0.813, 3.117), 0.176	
Other	0.597 (0.102, 3.48), 0.566	
**How often do you engage in recreational physical activity?**		
Never	1	
Rarely	1.006 (0.443, 2.287), 0.988	
1–2 times per week	1.396 (0.564, 3.456), 0.471	
3+ times per week	1.615 (0.538, 4,853), 0.393	
**How often are you in contact with nature?**		
Never	1	
Rarely	0.945 (0.375, 2.383), 0.905	
1–2 times per week	1.375 (0.495, 3.821), 0.541	
3+ times per week	2.5 (0.389, 16.049), 0.334	
**Negative impact of physical health on your ability to focus, learn and do well in university**		
A great deal	1	
Some	0.63 (0.252, 1.576), 0.324	
Not too much	0.836 (0.353, 1.979), 0.683	
Not at all	1.11 (0.456, 2.698), 0.818	
Not sure	0.4 (0.082, 1.942), 0.256	
**Have you engaged in internships, or other opportunities to gain practical experience in your field?**		
Yes	1	
No	0.624 (0.38, 1.023), 0.061	
**Are you actively involved in ECA?**		
Yes	1	
No	0.66 (0.397, 1.098), 0.11	
**Have you ever thought of dropping from university?**		
Yes	1	
No	1.418 (0.776, 2.589), 0.256	
**Will you finish your study on time?**		
Yes	1	1
No	0.622 (0.338, 1.146), 0.128	0.462 (0.21, 1.019), 0.056
Not sure	**0.47 (0.243, 0.906), 0.024**	0.574 (0.241, 1.368), 0.21
**Are you satisfied with the quality of education and the learning environment provided by your institution?**		
Yes	1	1
No	**0.547 (0.325, 0.922), 0.023**	**0.439 (0.207, 0.934), 0.033**
**Are you satisfied with the quality of services provided by your student affairs at your institution?**		
Yes	1	
No	1.092 (0.67, 1.778), 0.724	
**BMI**	1.044 (0.97, 1.124), 0.251	

Estimates shown in bold are those that are statistically significant at *p* < 0.05.

## Data Availability

The data presented in this study are available on request from the corresponding author. The data are not publicly available due to the privacy of the participants and ethical concerns.

## Supplementary Materials

The following supporting information can be downloaded at: https://www.mdpi.com/article/10.3390/ijerph21050597/s1, Supplementary File S1: Consent form; Supplementary File S2: The questionnaire.
