# Supplementary material for: Assessing the Relationship between Physical Health, Mental Health and Students’ Success among Universities in Lebanon: A Cross-Sectional Study
Source: Int J Environ Res Public Health. 2024 May 5;21(5):597. doi: 10.3390/ijerph21050597 (PMC11121208; doi:10.3390/ijerph21050597)
Supplement: Supplementary file 1 [file ijerph-21-00597-s001.zip › ijerph-2907909-supplementary.pdf]

International Journal of
Environmental Research and Public Health

MDPI

Article

Assessing the ~~Impact~~ Relationship between~~of~~ Physical Health, ~~and~~ Mental Health and~~on~~ Students' Success among Universities in Lebanon: A Cross-Sectional Study

Samer A. Kharroubi [1,2,*], Nayla Al-Akl [1,3], Sarah-joe Chamate [1], Tarek Abou Omar [1] and Rouba Ballout [2]

[1] Office of Student Affairs, American University of Beirut, P.O. BOX 11-0236, Riad El Solh, Beirut 1107-2020, Lebanon; sk157@aub.edu.lb (S.A.K.); na143@aub.edu.lb (N.A.); sc50@aub.edu.lb (S.C.); ta57@aub.edu.lb (T.A.)
[2] Department of Nutrition and Food Sciences, Faculty of Agricultural and Food Sciences, American University of Beirut, P.O. BOX 11-0236, Riad El Solh, Beirut 1107-2020, Lebanon; rb166@aub.edu.lb (R.B.); sk157@aub.edu.lb (S.A.K.)
[3] Department of Landscape Design and Ecosystem Management, Faculty of Agricultural and Food Sciences, American University of Beirut, P.O. BOX 11-0236, Riad El Solh, Beirut 1107-2020, Lebanon; na143@aub.edu.lb (N.A.)
* Correspondence: sk157@aub.edu.lb

Abstract: Background: Achieving high academic success is known to be influenced by many factors including, but not limiting to, physical and mental health. The present study aimed to assess the relationship~~impact~~ between~~of~~ physical ~~and~~ health, mental health and~~on~~ university students' success, and to explore the associations between these factors and their academic achievement; Methods: A cross-sectional, self-administered online survey was used to collect data from college students in three different universities in Lebanon during the Fall 2023 semester. Mental health was evaluated using validated screening tools for depression, anxiety, and stress, specifically the Patient Health Questionnaire (PHQ-9), the General Anxiety Disorder (GAD-7), and Cohen's Perceived Stress Scale (PSS), respectively. Additionally, general questions regarding physical health and lifestyle factors were incorporated into the questionnaire. Academic achievement was measured using students' grade point average (GPA); Results: A total of 261 students completed the self-administered online survey. Results revealed that approximately 42% and 36% of students were experiencing moderate to severe symptoms of depression and anxiety, respectively, and 75.1% of students exhibited symptoms of moderate stress. The majority of participants (99.2%) did not report any physical disability. Chi-square analysis revealed a significant association between mental health status (depression, anxiety, and stress) and GPA level (p=0.03, p=0.044, p=0.015, respectively). Multiple logistic regression models identified eight correlates of GPA and highlighted the relationship~~impact~~ between~~of~~ physical health and~~on~~ student success. For instance, students who considered themselves moderately active had lower odds of achieving a higher GPA than those who considered themselves active (OR=0.41, p=0.045); Conclusions: This is the first investigation on a Lebanese university students' academic success in relation to lifestyle and mental health profiles. The findings indicate that implementing public health programs and interventions targeting mental health and lifestyle behaviors is essential for enhancing student success.

Keywords: university students; student success; mental health; physical health; Lebanon

Citation: To be added by editorial staff during production.

Academic Editor: Firstname Lastname

Received: date
Revised: date
Accepted: date
Published: date

Copyright: © 2024 by the authors. Submitted for possible open access publication under the terms and conditions of the Creative Commons Attribution (CC BY) license (https://creativecommons.org/licenses/by/4.0/).

1. Introduction

University students play a crucial role in advancing knowledge and fostering progress in society. As future professionals, researchers, leaders, and policymakers, they hold the potential to positively impact the world [1]. Academic achievement, a concept that is essential to student's future success, is defined as the degree to which the students meet

their academic objectives [2]. According to the Cambridge University Reporter, examination performance is commonly used to assess academic performance [3]. In this study, academic achievement was measured by GPA, Unlike many similar studies, which mainly used subjective measures of academic performance, in our study, academic achievement was measured by GPA [3], which enhances the reliability of our findingsreflecting students' performance in assignments, tests, and exams. A higher score indicates better academic performance [2]. It is notable that students vary in their academic performance due to several factors, considering the numerous challenges faced by college students [44]. Addressing these factors could contribute to enhancing students' success.

Poor mental health among college students is becoming an increasing concern for public health and policy [5,65]. The rise in mental illness, particularly among university students, emphasizes the need to comprehend risk factors and potential solutions for an environment that supports mental health. Depression, a major mental health problem, has a remarkable impact on student's ability to perform life activities [76]. Therefore, it is necessary to explore the association between mental health and student's productivity, academic performance, and success. Additionally, university students deal with unstable living conditions and make adaptations to changes in their environment, diet, and lifestyle [87]. A common phenomenon among college students is weight gain during their initial years. For example, Kasparek et al.'s study revealed an average weight gain of 1.3-3.1 kg among freshmen during their first term at university [98]. These unhealthy eating habits and sedentary lifestyle choices may have adverse effects on health in adulthood [10]. Moreover, many students deal with various physical illnesses, including chronic diseases and poor nutrition [11]. This emphasizes the significance of prioritizing physical well-being among university students and recognizing its association with academic success.

Formatted: Not Highlight

Formatted: Not Highlight

Lebanon, a small middle-income country in the Middle East & North Africa (MENA) region, is struggling with a significant crisis, particularly in the aftermath of the economic meltdown in 2019, the COVID-19 pandemic, and the Beirut Port explosion in 2020. It is also essential to consider Lebanon's distinct socio-cultural background, which is distinguished by its diverse population, encompassing various cultures, and religious affiliations [12]. –These crises, coupled with the country's chaotic history, have had a profound impact on the mental health and overall well-being of the population [139], as well as on the education and lifestyle of university students [140,151]. The study findings would be relevant for neighboring countries facing similar challenges. –A 2022 study on a sample of university students in Lebanon revealed alarming statistics, with 22.6% and 34.4% of students exhibiting severe symptoms of depression and anxiety, respectively [162]. Another study by Fawaz et al. showed that 17.9% of Lebanese students experienced mild depression, 13.8% moderate depression, and 1.7% severe depression. Additionally, 21.9% of students reported moderate anxiety, 6.3% severe anxiety, and 2.3% extreme anxiety [173]. Among pharmacy students in Lebanon, a separate study found that 41.8% experienced severe or extremely severe anxiety, 30.7% reported depression, 28.5% faced stress, 27.7% dealt with moderate/severe insomnia, and 45.5% exhibited symptoms of PTSD [184].

It is also essential to consider Lebanon's distinct socio-cultural background, which is distinguished by its diverse population, encompassing various cultures, and religious affiliations [15]. To date, only one study explored the association between health behaviors, mental health, and academic achievement [196]. However, academic success was measured using the Subjective Academic Achievement Scale (SAAS). The findings indicated a significant association between a higher frequency of dining out and increased psychological distress with lower SAAS scores. To our knowledge, there is a gap in research regarding the correlation between mental and physical health and GPA among college students in Lebanon, as no previous studies have examined this.

Thus, the present study aims to address this research gap in Lebanon. This approach will provide a more objective assessment of students' performance. –The key objective of the study is to assess the impact ofrelationship between physical health, and mental health

andon university students' success, and to explore the associations between sociodemographic factors and academic achievement.

2. Materials and Methods

2.1. Study design and sampling

A cross-sectional study consisting of a random sampling procedure was conducted during the Fall semester of 2023 (October–December), involving students from three different universities across Lebanon. These universities included two renowned private institutions, the American University of Beirut (AUB) and the University of Balamand (UOB), as well as the Lebanese University, which is the sole public university with multiple branches and majors throughout the country. Based on a similar study conducted by Baert et al [2017], the required sample size was determined to be 220, with a 95% confidence interval and a precision level of 40%. To accommodate an additional 20% refusal rate, a total of 264 college students were included in the study. The sample size was stratified according to university size and gender, resulting in the need for a total of 137 women and 127 men for the study.

2.2. Data collection

After obtaining approval from the Dean of the Student Affairs Office at the respective universities (to avoid any undue influence or coercion), the research team visited the universities included in the study. They randomly approached students and invited them to participate in a self-administered online questionnaire. Upon agreeing to take part in the study and reviewing the consent form (Appendix A), participants proceeded to complete the survey (Appendix B). The survey's completion took approximately 10 minutes.

Participation in the survey was entirely optional and anonymous. Additionally, participants were encouraged to ask questions related to the study or seek additional clarification before consenting to participate. Moreover, the study received approval from the Institutional Review Board (IRB) at AUB, and the research team was Collaborative Institutional Training Initiative (CITI)-certified.

2.3. Survey format

The survey aimed to assess the mental and physical health of university students, along with factors associated with student success. According to the Cambridge University Reporter, examination performance is commonly used to assess academic performance [21]. In this study, academic achievement was measured by GPA, reflecting students' performance in assignments, tests, and exams. A higher score indicates better academic performance [2]. The questionnaire was created based on similar studies in the literature [162,2218] and comprised four sections. The first section consisted of questions about participants' socio-demographic characteristics and university-related factors, including age, gender, area of residency, educational level, and major.

Section 2 included validated and reliable scales that are used to assess mental health on an ordinal scale: the Patient Health Questionnaire (PHQ-9) for depression, the General Anxiety Disorder (GAD-7) for anxiety, and Cohen's Perceived Stress Scale (PSS) for stress. This section also encompassed some questions related to social support. The PHQ-9 comprises 9 statements on a 4-point Likert-type scale ranging from 0 (not at all) to 3 (nearly every day). For each respondent, the assigned values for PHQ-9 items were added to create a score ranging from 0 to 27. The resulting score was utilized to categorize individuals into different levels of depression: minimal (0–4), mild (5–9), moderate (10–14), moderately severe (15–19), and severe depression (20-27) [2319]. The GAD-7 is a 7-item self-reported anxiety scale, where each item is graded on the Likert scale from 0 (not at all) to 3 (nearly every day). For each respondent, the assigned values for GAD-7 items were summed to create a score ranging from 0 to 21. This score was used to classify individuals into categories of minimal (0–4), mild (5–9), moderate (10–14), and severe anxiety (15-21),

respectively [24~~0~~]. The PSS consists of 10 items, with responses rated on the Likert scale from 0 (never) to 4 (very often). For questions 4, 5, 7, and 8, the assigned values were reversed (i.e., 0 = 4, 1 = 3, 2 = 2, 3 = 1, 4 = 0). For each respondent, the assigned values were then summed. The total scores of the PSS ranged from 0 to 40, with scores of 0-13 representing low stress, 14-26 indicating moderate stress, and 27-40 signifying high perceived stress [25~~1~~].

The third section focused on the participants' physical health (for instance BMI…) and lifestyle factors (for instance physical activity, sleeping habit, diet…) whereas the last section included questions on students' success. The questionnaire was pilot tested on 10 students to check for clarity. Data collected during the pilot testing phase were not incorporated into the present study. Appendix B contains a copy of the questionnaire used for data collection.

2.4. Statistical analysis

Data were rigorously checked for completeness and were then entered into the Statistical Package for the Social Sciences (SPSS) version 29.0 (SPSS Inc., Chicago, IL, USA) for data analysis. Descriptive statistics were used, such as counts and percentages for the categorical variables and ~~as~~ means and standard deviations (SD) for the continuous ones. Chi-square ($\chi 2$) was used to calculate the association between two categorical variables. Note that GPA for UOB students was multiplied by 0.98, whereas for LU students by 1.2 in accordance to AUB equivalence system. GPA was then dichotomized. The mean GPA score was considered the cut-point. Participants reporting a GPA below the mean score, i.e. between 0-79 were considered to have a low to moderate GPA, and those reporting a GPA above or equal to the mean score, i.e. ~~of~~ 80 and above were considered to have a high GPA.

Simple and multiple logistic regression were applied to investigate which factors were associated with student success, using the GPA score as dependent variable and the sociodemographic factors, such as mental and physical health, and university as independent variables. All variables that were found significant in the simple analysis were added simultaneously to the multiple regression models as independent variables. Results from the logistic regression analyses were expressed as odds ratios (OR) with their respective 95% confidence intervals (CI). A p-value below 0.05 was considered statistically significant in all analyses.

3. Results

3.1. Sociodemographic characteristics

A total of 264 students completed the online survey, with 261 providing complete data and being included in the final analysis (resulting in a 99% completion rate). The sociodemographic characteristics of the study population, along with some information about their studies, are presented in Table 1. Based on the gender and university stratification plan, approximately 52% of the students were women, with 27.6% enrolled in AUB, 51.3% in LU, and 21.1% in UOB.

The average age of college students was 20.29 years (SD=2.025), ranging between 17 and 33 years old. The majority of students were from Beirut (40.2%) and Mount-Lebanon (31%). A significant portion of the participants were Lebanese (92.3%), and many were enrolled in non-health-related majors (73.6%). About 64.4% of the participants lived with their families. Additionally, around 36% of the students reported a personal monthly income between 100-300 USD. Nearly half of the participants' parents held a university degree. Most students had working fathers (92.3%), while 56.7% had working mothers. Moreover, more than two-thirds of the participants had no employment (72.4%) and did not receive any financial aid or scholarship (67%). Approximately 32% reported studying more than 30 hours per week. Finally, 48.7% of college students had a low to moderate GPA, while 51.3% had a ~~moderate to~~ high GPA.

Table 1. Sociodemographic characteristics [a] and university factors [b] of college students in Lebanon in the study sample (n=261).

Characteristics		n (%)	Low to moderate GPA n (%)	High GPA n (%)	P-value
Gender [a]	Man	126 (48.3)	63 (50)	63 (50)	0.675
	Women	135 (51.7)	64 (47.4)	71 (52.6)	
Age [a]	Mean: 20.3	SD: 2			
Area of residency [a]	Beirut	105 (40.2)	45 (52.9)	60 (57.1)	**0.006**
	Mount-Lebanon	81 (31)	53 (65.4)	28 (34.6)	
	South	29 (11.1)	13 (44.8)	16 (55.2)	
	North	28 (10.7)	10 (35.7)	18 (64.3)	
	Bekaa	18 (6.9)	6 (33.3)	2 (66.7)	
Nationality [a]	Lebanese	241 (92.3)	114 (47.3)	127 (52.7)	0.128
	Non-Lebanese	20 (7.7)	13 (65)	7 (35)	
Stage of study [b]	1st semester	48 (18.4)	26 (54.2)	22 (45.8)	0.258
	2nd – 3rd semester	65 (24.9)	33 (50.8)	32 (49.2)	
	4th - 5th semester	59 (22.6)	30 (50.8)	29 (49.2)	
	6th - 8th semester	56 (21.5)	28 (50)	28 (50)	
	9th + semester	33 (12.6)	10 (30.3)	23 (69.7)	
Major [b]	Non-Health related	192 (73.6)	101 (52.6)	91 (47.4)	**0.033**
	Health-related	69 (26.4)	26 (37.7)	43 (62.3)	
Where do you live during university years? [a]	Family	168 (64.4)	86 (51.2)	82 (48.8)	0.542
	Roommates	72 (27.5)	32 (44.4)	40 (55.6)	
	Alone	21 (8)	9 (42.9)	12 (57.1)	
Personal monthly income/allowance (USD) [a]	<100	82 (31.4)	42 (51.2)	40 (48.8)	0.137
	100-300	93 (35.6)	45 (48.4)	48 (51.6)	
	300-500	49 (18.8)	28 (57.1)	21 (42.9)	
	≥500	37 (14.2)	12 (32.4)	25 (67.6)	
Monthly income of household (USD) [a]	<500	38 (14.6)	17 (44.7)	21 (55.3)	0.822
	500-800	50 (19.2)	27 (54)	23 (46)	
	800-1000	69 (26.4)	34 (49.3)	35 (50.7)	
	≥1000	104 (39.8)	49 (47.1)	55 (52.9)	
Education of father [a]	Intermediate or less	41 (15.7)	18 (43.9)	23 (56.1)	0.824
	High school	66 (25.3)	35 (53)	31 (47)	
	University	137 (52.5)	66 (48.2)	71 (51.8)	
	Other	17 (6.5)	8 (47.1)	9 (52.9)	
Education of mother [a]	Intermediate or less	26 (9.9)	14 (53.8)	12 (46.2)	0.719
	High school	66 (25.3)	35 (53)	31 (47)	
	University	149 (57.1)	68 (45.6)	81 (54.4)	
	Other	20 (7.7)	10 (50)	10 (50)	
Father's employment status [a]	Not working	20 (7.7)	8 (40)	12 (60)	0.703

	Self-employed	97 (37.2)	47 (48.5)	50 (51.5)	
	Employed	144 (55.1)	72 (50)	72 (50)	
Mother's employment status [a]	Not working	148 (56.7)	77 (52)	71 (48)	**0.025**
	Self-employed	23 (8.8)	5 (21.7)	18 (78.3)	
	Employed	90 (38.5)	45 (50)	45 (50)	
Your current job [a]	No job	189 (72.4)	87 (46)	102 (54)	0.169
	Employed	72 (27.6)	40 (55.6)	32 (44.4)	
Time spent on study (per week) [b]	<10h	34 (13)	14 (41.2)	20 (58.8)	0.217
	10h-19h	70 (26.8)	41 (58.6)	29 (41.4)	
	20h-29h	74 (28.4)	36 (48.6)	38 (51.4)	
	30+h	83 (31.8)	36 (43.4)	47 (56.6)	
Receive financial aid or scholarship? [b]	Yes	86 (33)	41 (47.7)	45 (52.3)	0.823
	No	175 (67)	86 (49.1)	89 (50.9)	
Do you agree that your life is stressful? [a]	1 (Strongly disagree)	7 (2.7)	3 (42.9)	4 (57.1)	0.094
	2	33 (12.6)	15 (45.5)	18 (54.5)	
	3	72 (27.6)	26 (36.1)	46 (63.9)	
	4	85 (32.6)	46 (54.1)	39 (45.9)	
	5 (Strongly agree)	64 (24.5)	37 (57.8)	27 (42.2)	
University enrolled in [b]	American Universi~~sri~~ty of Beirut	72 (27.6)	30 (41.7)	42 (58.3)	**0.017**
	Lebanese University	134 (51.3)	61 (45.5)	73 (54.5)	
	University of Balamand	55 (21.1)	36 (65.5)	19 (34.5)	
GPA[1] [b]	Low to moderate	127 (48.7)			
	High	134 (51.3)			
BMI[2] [a]	Mean: 22.4	SD: 3.4			

3.2. *Mental health of participants and its association with GPA*

Among the study sample (Table 2), 73.5% of students exhibited mild to moderately severe symptoms of depression, and 80.5% displayed mild to severe symptoms of anxiety. Specifically, 44.1% showed mild symptoms of anxiety, while 10.7% exhibited severe symptoms. Approximately 75.1% presented with moderate symptoms of stress, and 13.4% of students exhibited high perceived stress. More than half of the participants (64.7%) believed that their mental health had a negative impact on their ability to learn, focus, and perform well in university.

As indicated in Table 2, significant differences were observed in the mental health status (depression, anxiety, and stress) of college students based on their GPA level. The rating of the negative impact of mental health was also significantly associated with GPA level.

[1] GPA: Grade Point Average

[2] BMI: Body Mass Index

Table 2. Mental Health of college students (n=261).

Characteristics		n (%)	Low to moderate GPA n (%)	High GPA n (%)	P-value
Patient Health Questionnaire (PHQ9)	Minimal depression [1-4]	52 (19.9)	17 (32.7)	35 (67.3)	**0.03**
	Mild depression [5-9]	98 (37.5)	51 (52)	47 (48)	
	Moderate depression [10-14]	51 (19.5)	23 (45.1)	28 (54.9)	
	Moderately severe depression [15-19]	43 (16.5)	28 (65.1)	15 (34.9)	
	Severe depression [20+]	17 (6.5)	8 (47.1)	9 (52.9)	
General Anxiety Disorder (GAD7)	Minimal anxiety [0-4]	51 (19.5)	17 (33.3)	34 (66.7)	**0.044**
	Mild anxiety [5-9]	115 (44.1)	56 (48.7)	59 (51.3)	
	Moderate anxiety [10-14]	67 (25.7)	40 (59.7)	27 (40.3)	
	Severe anxiety [15+]	28 (10.7)	14 (50)	14 (50)	
Cohen's Perceived Stress Scale (PSS)	Low stress [0-13]	30 (11.5)	14 (46.7)	16 (53.3)	**0.015**
	Moderate stress [14-26]	196 (75.1)	88 (44.9)	108 (55.1)	
	High perceived stress [27-40]	35 (13.4)	25 (71.4)	10 (28.6)	
Number of people that support you when you feel down	0	14 (5.4)	6 (42.9)	8(57.1)	0.089
	1	87 (33.3)	52 (59.8)	35 (40.2)	
	2-3	97 (37.2)	41 (42.3)	56 (57.7)	
	>3	63 (24.1)	28 (44.4)	35 (55.6)	
Satisfaction with this support	1 (very unsatisfied)	15 (5.7)	9 (60)	6(40)	0.126
	2	23 (8.8)	16 (69.6)	7 (30.4)	
	3	67 (25.7)	35 (52.2)	32 (47.8)	
	4	76 (29.1)	33 (43.4)	43 (56.6)	
	5 (very satisfied)	80 (30.7)	34 (42.5)	46 (57.5)	
Negative impact of mental health on your ability to focus, learn and do well in university	A great deal	76 (29.1)	48 (63.2)	28 (36.8)	**0.035**
	Some	93 (35.6)	40 (43)	53 (57)	
	Not too much	69 (26.4)	27 (39.1)	42 (60.9)	
	Not at all	18 (6.9)	9 (50)	9 (50)	
	Not sure	5 (1.9)	3 (60)	2 (40)	

3.3. Physical health of participants and its association with GPA

As shown in Table 3, the majority of college students in the study sample did not report any physical disability (99.2%) or chronic disease (83.1%). Among those who reported chronic disease, asthma was the most prevalent (38.6%). About 52.1% of participants experienced frequent headaches, fatigue, or stomachaches. Only 36.4% considered themselves physically active, while 45.6% reported being moderately active, and 18% described themselves as sedentary. Moreover, 47.9% of students went to the gym, 38.3% were daily smokers, and 33.3% consumed alcohol, with 24.5% doing so less than 2 times per week. Approximately 14.2% of participants identified as underweight, while 11.5% considered themselves obese. More than half of the participants (59.4%) were satisfied with their weight, and 52.5% reported weight fluctuations during their university years. Additionally, 65.9% of students reported having regular sleeping habits, and 57.5% believed they followed healthy eating habits, with homemade food being the most preferred option (42.5%). Only 32.9% of participants believed that their physical health negatively impacted their ability to learn, focus, and perform well in university. Finally, GPA was significantly associated with physical activity, smoking habits, gym attendance, and weight satisfaction.

Table 3. Physical health of college students (n=261).

Characteristics		n (%)	Low to moderate GPA n (%)	High GPA n (%)	P-value
Do you suffer from any physical disability?	Yes	2 (0.8)	2 (100)	0 (0)	0.145
	No	259 (99.2)	125 (48.3)	134 (51.7)	
Do you suffer from any chronic disease?	Yes	44 (16.9)	23 (52.3)	21 (47.7)	0.599
	No	217 (83.1)	104 (47.9)	113 (52.1)	
Which chronic disease do you suffer from? (n=44)	Asthma	17 (38.6)	-	-	
	Obesity	3 (6.8)			
	Hypertension	4 (9)			
	Poor oral health	1 (2.3)			
	Digestive system diseases	7 (15.9)			
	Diabetes	5 (11.4)			
	Muscle pains	2 (4.5)			
	Other	5 (11.4)			
Do you suffer frequently from headaches, stomachaches, fatigue?	Yes	136 (52.1)	71 (52.2)	65 (47.8)	0.232
	No	125 (47.9)	56 (44.8)	69 (55.2)	
Physical activity	Active	95 (36.4)	35 (36.8)	60 (63.2)	**0.012**
	Moderately active	119 (45.6)	64 (53.8)	55 (46.2)	
	Sedentary	47 (18)	28 (59.6)	19 (40.4)	
Do you go to the gym?	Yes	125 (47.9)	49 (39.2)	76 (60.8)	**0.003**
	No	136 (52.1)	78 (57.4)	58 (42.6)	
Are you a daily smoker?	Yes	100 (38.3)	59 (59)	41 (41)	**0.008**
	No	161 (61.7)	68 (42.2)	93 (57.8)	
What do you smoke? (n=100)	Cigarettes	49 (49)	-	-	
	Water-pipe	27 (27)			
	Vape	19 (19)			
	All the above	4 (4)			
	Other	1(1)			
Alcohol use	<2 times per week	64 (24.5)	29 (45.3)	35 (54.7)	0.924
	2 times per week	13 (5)	7 (53.8)	6 (46.2)	
	>2 times per week	10 (3.8)	5 (50)	5 (50)	
	I don’t drink alcohol	174 (66.7)	86 (49.4)	88 (50.6)	
How do you describe your weight according to BMI?	Under weight	37 (14.2)	20 (54.1)	17 (45.9)	0.449
	Normal	194 (74.3)	90 (46.4)	104 (53.6)	
	Overweight/ Obese	30 (11.5)	17 (56.7)	13 (43.3)	
Are you satisfied with your weight?	Yes	155 (59.4)	67 (43.2)	88 (56.8)	**0.034**
	No	106 (40.6)	60 (56.6)	46 (43.4)	
Did you lose/gain any weight during your university years?	Lose	68 (26.1)	36 (52.9)	32 (47.1)	0.704
	Gain	69 (26.4)	32 (46.4)	37 (53.6)	
	No	124 (47.5)	59 (47.6)	65 (52.4)	

Daily sleep hours	≤6	65 (24.9)	30 (46.2)	35 (53.8)	0.418
	7	61 (23.4)	28 (45.9)	33 (54.1)	
	8	66 (25.3)	38 (57.6)	28 (42.4)	
	>8	69(26.4)	31 (44.9)	38 (55.1)	
Sleeping habit	Regular	172 (65.9)	83 (48.3)	89 (51.7)	0.856
	Irregular	89 (34.1)	44 (49.4)	45 (50.6)	
Do you consider yourself to follow healthy eating habits?	Yes	150 (57.5)	70 (46.7)	80 (53.3)	0.454
	No	111 (42.5)	57 (51.4)	54 (48.6)	
Which option best describes your diet?	Mostly fast/processed food	68 (26.1)	37 (54.4)	31 (45.6)	0.22
	Mostly homemade food	111 (42.5)	51 (45.9)	60 (54.1)	
	Mostly vegan or vegetarian	6 (2.3)	5 (83.3)	1 (16.7)	
	All the above	70 (26.8)	30 (42.9)	40 (57.1)	
	Other	6 (2.3)	4 (66.7)	2 (33.3)	
How often do you engage in recreational physical activity?	Never	27 (10.3)	14 (51.9)	13 (48.1)	0.561
	Rarely	147 (56.3)	76 (51.7)	71 (48.2)	
	1-2 times per week	62 (23.8)	27 (43.5)	35 (56.5)	
	3+ times per week	25 (9.6)	10 (40)	15 (60)	
How often are you in contact with nature?	Never	20 (7.7)	10 (50)	10(50)	0.462
	Rarely	177 (67.8)	91 (51.4)	86 (48.6)	
	1-2 times per week	57 (21.8)	24 (42.1)	33 (57.9)	
	3+ times per week	7 (2.7)	2 (28.6)	5 (71.4)	
Negative impact of physical health on your ability to focus, learn and do well in university	A great deal	27 (10.3)	12 (44.4)	15 (55.6)	0.41
	Some	59 (22.6)	33 (55.9)	26 (44.1)	
	Not too much	92 (35.2)	45 (48.9)	47 (51.1)	
	Not at all	74 (28.4)	31 (41.9)	43 (58.1)	
	Not sure	9 (3.4)	6 (66.7)	3 (33.3)	

3.4. University factors and their association with GPA

As shown in Table 4, a significant portion of the participants (79.3%) never thought of dropping out of university. More than half of them (59.4%) believed they would complete their studies on time. Approximately 40% engaged in internships or other opportunities to gain practical experience in their fields and actively participated in extracurricular activities (ECA). Furthermore, 66.7% of students expressed satisfaction with the quality of education and the learning environment provided by their institution, while 54.8% were pleased with the quality of services offered by the student affairs office at their institution. Finally, the results revealed a significant association between GPA and the likelihood of finishing studies on time, as well as satisfaction with the quality of education provided by the institution.

Table 4. University factors (n=261).

**Characteristics**		**n (%)**	**Low to moderate GPA n (%)**	**High GPA n (%)**	**P-value**
Have you ever thought of dropping from university?	Yes	54 (20.7)	30 (55.6)	24(44.4)	0.255
	No	207 (79.3)	97 (46.9)	110 (53.1)	
	Yes	155 (59.4)	66 (42.6)	89(57.4)	**0.046**

Will you finish your study on time?	No,<br>Not sure	57 (21.8)<br>49 (18.8)	31 (54.4)<br>30 (61.2)	26 (45.6)<br>19 (38.8)	
Are you satisfied with the quality of education and the learning environment provided by your institution?	Yes<br>No	174 (66.7)<br>87 (33.3)	76 (43.7)<br>51 (58.6)	98(56.3)<br>36 (41.4)	**0.023**
Are you satisfied with the quality of services provided by your student affairs at your institution?	Yes<br>No	143 (54.8)<br>118 (45.2)	71 (49.7)<br>56 (47.5)	72(50.3)<br>62 (52.5)	0.724
Have you engaged in internships, or other opportunities to gain practical experience in your field?	Yes<br>No	112 (42.9)<br>149 (57.1)	47 (42)<br>80 (53.7)	65(58)<br>69 (46.3)	0.061
Are you actively involved in Extracurricular activities?	Yes<br>No	95 (36.4)<br>166 (63.6)	40 (42.1)<br>87 (52.4)	55(57.9)<br>79 (47.6)	0.109

3.5. Simple and multiple logistic regression analyses

Simple logistic regression analysis revealed that fifteen predictors were significantly associated with participants' GPA level (Table 5). These predictors included the area of residency (OR = 0.396, p = 0.002), where participants from Mount Lebanon were less likely to have a better GPA than those residing in Beirut. Other significant predictors comprised the stage of study (OR = 2.718, p = 0.036), major (OR = 1.836, p = 0.035), mother's employment status (OR = 3.904, p = 0.01), university enrollment (OR = 0.377, p = 0.009), PHQ9 (Mild depression: OR = 0.448, p = 0.025; Moderately severe depression: OR = 0.26, p = 0.002), GAD7 (OR = 0.338, p = 0.005), PSS (OR = 0.35, p = 0.045), Negative impact of mental health (A great deal: OR = 2.271, p = 0.01; Some: OR = 2.667, p = 0.004), Physical activity (Moderately active: OR = 0.501, p = 0.014; Sedentary: OR = 0.396, p = 0.011), Gym (OR = 0.479, p = 0.004), Smoking (OR = 1.968, p = 0.009), Weight satisfaction (OR = 0.584, p = 0.034), Finishing study on time (OR = 0.47, p = 0.024), and Satisfaction with the quality of education provided by the university (OR = 0.547, p = 0.023).

Results from multiple logistic analysis revealed several significant associations with participants' GPA levels. More specifically, participants with a health-related major were more likely to have a high GPA compared to those with a non-health major (OR=3.874, p=0.007). Students studying at UOB had lower odds of having a high GPA compared to those studying at AUB (OR=0.222, p=0.004). Additionally, participants who reported being moderately active were less likely to have a high GPA compared to those who reported being active (OR=0.41, p=0.045). Students who don't smoke daily were more likely to have a high GPA compared to those who smoke daily (OR=2.948, p=0.003). Further, participants who reported being unsatisfied with the quality of education and the learning environment provided by their institution were less likely to have a high GPA compared to those who reported being satisfied (OR=0.439, p=0.033). Also, students who have working mothers specifically self-employed were more likely to have a high GPA compared to those who have non-working mothers (OR= 3.971, p=0.043). Finally, results showed that the

student's area of residency, and whether they consider that mental health has a negative impact on their studies were all significantly associated with their GPA level (OR=0.363, p=0.011; OR=3.019, p=0.038, respectively).

Table 5. Simple and Multiple Logistic regression.

	**GPA**	
	Simple<br>OR, (95% CI), p-Value	Multiple<br>OR, (95% CI), p-Value
**Age**	1 (0.887, 1.128), 0.999	
**Gender**		
Male	1	
Female	1.109 (0.682, 1.803), 0.675	
**Nationality**		
Lebanese	1	
Non-Lebanese	0.483 (0.186, 1.254), 0.135	
**Area of residency**		
Beirut	1	1
Mount-Lebanon	**0.396 (0.218, 0.721), 0.002**	**0.363 (0.166, 0.791), 0.011**
South	0.923 (0.403, 2.112), 0.850	0.912 (0.309, 2.691), 0.867
North	1.35 (0.569, 3.204), 0.496	1.201 (0.374, 3.859), 0.758
Bekaa	1.5 (0.523, 4.301), 0.451	0.603 (0.147, 2.485), 0.484
**Stage of study**		
1st semester	1	1
2nd – 3rd semester	1.146 (0.543, 2.42), 0.721	1.108 (0.397, 3.095), 0.845
4th - 5th semester	1.142 (0.532, 2.451), 0.732	1.225 (0.448, 3.353), 0.693
6th - 8th semester	1.182 (0.546, 2.559), 0.672	1.071 (0.377, 3.041), 0.897
9th + semester	**2.718 (1.068, 6.921), 0.036**	1.558 (0.432, 5.614), 0.498
**Major**		
Non-health related	1	1
Health-related	**1.836 (1.045, 3.224), 0.035**	**3.874 (1.455, 10.321), 0.007**
**Where do you live during your university years?**		
Family	1	
Roommates	1.311 (0.753, 2.283), 0.339	
Alone	1.398 (0.56, 3.494), 0.473	
**Personal monthly income/allowance (USD)**		
<100	1	
100-300	1.12 (0.618, 2.029), 0.708	
300-500	0.788 (0.386, 1.606), 0.511	
≥500	2.187 (0.97, 4.933), 0.059	
**Monthly income of household**		
<500	1	
500-800	0.69 (0.295, 1.609), 0.39	
800-1000	0.833 (0.376, 1.845), 0.653	
≥1000	0.909 (0.431, 1.917), 0.801	
**Education of father**		
Intermediate or less	1	
High school	0.693 (0.317, 1.518), 0.359	
University	0.842 (0.417, 1.699), 0.631	
Other	0.88 (0.283, 2.738), 0.826	
**Education of mother**		
Intermediate or less	1	
High school	1.033 (0.416, 2.567), 0.944	
University	1.39 (0.603, 3.205), 0.44	

Other	1.167 (0.363, 3.749), 0.796	
**Father's employment status**		
Not working	1	
Self-employed	0.709 (0.266, 1.888), 0.492	
Employed	0.667 (0.257, 1.728), 0.404	
**Mother's employment status**		
Not working	1	1
Self-employed	**3.904 (1.377, 11.068), 0.01**	**3.971 (1.043, 15.116), 0.043**
Employed	1.085 (0.642, 1.832), 0.762	1.315 (0.641, 2.698), 0.455
**Your current job**		
No job	1	
Employed	0.682 (0.395, 1.178), 0.17	
**Time spent on study**		
<10h	1	
10h-19h	0.495 (0.215, 1.138), 0.098	
20h-29h	0.739 (0.325, 1.68), 0.47	
30+h	0.914 (0.47, 2.053), 0.827	
**Receive financial aid or scholarship**		
Yes	1	
No	0.943 (0.562, 1.581), 0.823	
**Do you agree that your life is stressful?**		
1(Strongly disagree)	1	
2	0.9 (0.173, 4.669), 0.9	
3	1.327 (0.275, 6.393), 0.724	
4	0.636 (0.134, 3.016), 0.636	
5 (Strongly agree)	0.547 (0.113, 2.649), 0.454	
**University enrolled in**		
AUB	1	1
Lebanese University	0.855 (0.479, 1.525), 0.595	1.81 (0.752, 4.356), 0.186
Balamand	**0.377 (0.182, 0.78), 0.009**	**0.222 (0.080, 0.617), 0.004**
**PHQ9**		
Minimal depression [1-4]	1	1
Mild depression [5-9]	**0.448 (0.222, 0.903), 0.025**	0.652 (0.187, 2.278), 0.503
Moderate [10-14]	0.591 (0.266, 1,316), 0.198	1.891 (0.41, 8.714), 0.414
Moderately severe [15-19]	**0.26 (0.111, 0.611), 0.002**	1.457 (0.263, 8.064), 0.666
Severe depression [20+]	0.546 (0.179, 1.666), 0.288	3.119 (0.28, 34.785), 0.355
**GAD7**		
Minimal anxiety [0-4]	1	1
Mild anxiety [5-9]	0.527 (0.265, 1.048), 0.068	0.486 (0.128, 1.845), 0.289
Moderate [10-14]	**0.338 (0.158, 0.722),0.005**	0.344 (0.068, 1.733), 0.196
Severe anxiety [15+]	0.5 (0.195, 1.283), 0.149	0.58 (0.06, 5.59), 0.637
**PSS**		
Low stress [0-13]	1	1
Moderate stress [14-26]	1.074 (0.497, 2.321), 0.856	1.215 (0.295, 5), 0.788
High perceived stress [27-40]	**0.35 (0.125, 0.976), 0.045**	0.464 (0.072, 2.99), 0.42
**Number of people that support you when you feel down**		
0	1	
1	0.505 (0.161, 1.582), 0.241	
2-3	1.024 (0.33, 3.179), 0.967	
>3	0.938 (0.291, 3.019), 0.914	
**Satisfaction with this support**		
1 (very unsatisfied)	1	
2	0.656 (0.168, 2.563), 0.545	

3	1.371 (0.439, 4.283), 0.587	
4	1.955 (0.633, 6.04), 0.244	
5 (very satisfied)	2.029 (0.659, 6.245), 0.217	
**Negative impact of mental health on your ability to focus, learn and do well in university**		
A great deal	1	1
Some	**2.271 (1.221, 4.227), 0.01**	**3.019 (1.063, 8.573), 0.038**
Not too much	**2.667 (1.362, 5.219), 0.004**	2.688 (0.83, 8.702), 0.099
Not at all	1.714 (0.609, 4.825), 0.307	0.645 (0.087, 4.775), 0.668
Not sure	1.143 (0.18, 7.26), 0.887	0.227 (0.019, 2.7), 0.24
**Do you suffer from any physical disability?**		
Yes	1	
No	-	
**Do you suffer from any chronic disease?**		
Yes	1	
No	1.19 (0.622, 2.277), 0.599	
**Do you suffer frequently from headaches, stomachaches, fatigue?**		
Yes	1	
No	1.346 (0.827, 2.191), 0.232	
**Physical activity**		
Active	1	1
Moderately active	**0.501 (0.289, 0.87), 0.014**	**0.41 (0.171, 0.98), 0.045**
Sedentary	**0.396 (0.193, 0.81), 0.011**	0.594 (0.184, 1.915), 0.383
**Do you go to the gym?**		
Yes	1	1
No	**0.479 (0.292, 0.786), 0.004**	0.695 (0.321, 1.502), 0.355
**Are you a daily smoker?**		
Yes	1	1
No	**1.968 (1.186, 3.266), 0.009**	**2.948 (1.457, 5.961), 0.003**
**Alcohol use**		
<2 times per week	1	
2 times per week	0.71 (0.215, 2,349), 0.575	
>2 times per week	0.829 (0.218, 3.145), 0.782	
I don't drink alcohol	0.848 (0.477, 1.507), 0.574	
**How do you describe your weight?**		
Under weight	1	
Normal	1.359 (0.671, 2.753), 0.394	
Overweight/ Obese	0.9 (0.341, 2.372), 0.831	
**Are you satisfied with your weight?**		
Yes	1	1
No	**0.584 (0.355, 0.961), 0.034**	0.734 (0.379, 1.42), 0.358
**Did you lose/gain any weight during your university years?**		
Lose	1	
Gain	1.301 (0.665, 2.545), 0.443	
No	1.239 (0.685, 2.241), 0.478	
**Sleeping hours**		
≤6	1	
7	(0.501, 2.036), 0.977	

8	0.632 (0.317, 1.259), 0.192	
>8	1.051 (0.532, 2.075), 0.887	
**Sleeping habits**		
Regular	1	
Irregular	0.954 (0.572, 1.591), 0.856	
**Do you consider yourself to follow healthy eating habits?**		
Yes	1	
No	0.829 (0.507, 1.355), 0.454	
**Which option best describes your diet?**		
Mostly fast/processed food	1	
Mostly homemade food	1.404 (0.766, 2.574),0.272	
Mostly vegan or vegetarian	0.239 (0.026, 2.153), 0.202	
All the above	1.591 (0.813, 3.117), 0.176	
Other	0.597 (0.102, 3.48), 0.566	
**How often do you engage in recreational physical activity?**		
Never	1	
Rarely	1.006 (0.443, 2.287), 0.988	
1-2 times per week	1.396 (0.564, 3.456), 0.471	
3+ times per week	1.615 (0.538, 4,853), 0.393	
**How often are you in contact with nature?**		
Never	1	
Rarely	0.945 (0.375, 2.383), 0.905	
1-2 times per week	1.375 (0.495, 3.821), 0.541	
3+ times per week	2.5 (0.389, 16.049), 0.334	
**Negative impact of physical health on your ability to focus, learn and do well in university**		
A great deal	1	
Some	0.63 (0.252, 1.576), 0.324	
Not too much	0.836 (0.353, 1.979), 0.683	
Not at all	1.11 (0.456, 2.698), 0.818	
Not sure	0.4 (0.082, 1.942), 0.256	
**Have you engaged in internships, or other opportunities to gain practical experience in your field?**		
Yes	1	
No	0.624 (0.38, 1.023), 0.061	
**Are you actively involved in ECA?**		
Yes	1	
No	0.66 (0.397, 1.098), 0.11	
**Have you ever thought of dropping from university?**		
Yes	1	
No	1.418 (0.776, 2.589), 0.256	
**Will you finish your study on time?**		
Yes	1	1
No	0.622 (0.338, 1.146), 0.128	0.462 (0.21, 1.019), 0.056
Not sure	**0.47 (0.243, 0.906), 0.024**	0.574 (0.241, 1.368), 0.21

**Are you satisfied with the quality of education and the learning environment provided by your institution?**		
Yes	1	1
No	**0.547 (0.325, 0.922), 0.023**	**0.439 (0.207, 0.934), 0.033**
**Are you satisfied with the quality of services provided by your student affairs at your institution?**		
Yes	1	
No	1.092 (0.67, 1.778), 0.724	
**BMI**	1.044 (0.97, 1.124), 0.251	

4. Discussion

4.1. General findings

The present study is among the few conducted in the MENA region, including Lebanon, that investigates the correlations between mental health, physical health, and academic achievement. To the best of our knowledge, this is the first study in Lebanon to employ GPA as a measure of students' success, departing from more subjective methods such as the Subjective Academic Achievement Scale (SAAS) [19~~16~~].

Our results revealed alarming rates of depression and anxiety, surpassing those documented in previous studies. Specifically, our findings indicated that nearly 76% of students exhibited mild to moderately severe symptoms of de~~-~~pression, and approximately 70% reported mild to moderate symptoms of anxiety. In contrast, studies conducted in 2018 showed that 56% of students were experiencing mild to moderate depression symptoms, with 36% and 34% presenting combined symptoms of depression and anxiety, respectively [26~~2~~,27~~3~~]. Despite using identical mental health indicators, these variations may be attributed to methodological differences, particularly the focus on single universities in the latter studies. The ongoing crisis in the country could be another influencing factor contributing to the increased prevalence of mental health issues among young adults, especially since the previous studies were conducted before the economic crisis and the onset of the COVID-19 pandemic. Further~~-~~more, the levels of anxiety reported in our study were higher than those documented among college students in Lebanon in 2021 (50%) [16~~12~~], the United States (15.9%) [26~~2~~], and Canada (32.6%) [28~~4~~]. Additionally, approximately 75% of our study sample exhibited moderate stress levels, a figure higher than the reported levels among Pharmacy students in Lebanon (27.7%) [18~~14~~].

The present study also revealed that ~~over half of the students (64.7%)~~a significant percentage of students reported a negative impact of their mental health on academic performance. This finding is consistent with a Student Voice survey on health and wellness conducted by Inside Higher Ed and College Pulse, where half of the students asserted that their well-being adversely affects their academic progress [29~~5~~]. In a longitudinal study conducted in the United States, it was found that mental health problems predicted delayed academic success (GPA) [30~~26~~]. It is worth noting that mental health issues and their ~~impact on~~relationship with students' success have not received sufficient attention in the MENA region compared to other countries [31~~27~~]. The present study's findings highlight a significant association between mental health and student success. These findings highlight the urgent need for comprehensive mental health support services on university campuses to address the well-being of students and mitigate the adverse effects on their academic success.

Our findings indicate that only 11.5% of students perceive themselves as overweight or obese, a considerably lower proportion compared to other studies. For example, a cross-sectional study encompassing university students from 22 low, middle-income, and emerging economy countries reported that 22% of participants were overweight or obese

Formatted: MDPI_2.2_heading2

Formatted: Not Highlight

Formatted: Not Highlight

Formatted: Not Highlight

[32~~28~~]. Similarly, a study conducted in Egypt found that approximately one quarter of males and one third of females fell into the overweight/obese category [31~~27~~]. A potential explanation for our results may be attributed to self-reported bias, as students were required to assess their weight based on BMI. Additionally, the mean BMI of college students in our study sample was 22.41 ± 3.37, aligning with acceptable ranges observed in previous studies conducted in Lebanon, Morocco, and Saudi Arabia [19~~6~~,33~~29~~,34~~0~~,35~~1~~]. Although our study did not identify any association between BMI and GPA, it is noteworthy that several studies have indicated a negative ~~impact of high BMI on~~relationship between BMI and academic achievement [36~~2~~,37~~3~~]. Future studies should examine the complex relationship between academic achievement and weight status, accounting for variables like health behaviors and body image perception.

Our results indicated an association between GPA and the area of residency, contrary to findings in other studies. For instance, Alfifi et al. discovered that the residential area has no significant influence on academic achievement [38,39,40~~4~~]. In contrast, their research found that women outperformed male students, while our findings revealed no gender-based differences in GPA among college students in the study sample. Additionally, in line with previous studies, our results demonstrated that students pursuing health-related majors were more likely to achieve higher GPAs compared to non-health ~~students~~ majors [39~~5~~,40~~36~~]. This differs from a study conducted in Lebanon, where better academic achievement was observed among students with non-scientific majors, potentially influenced by the subjective method used to measure academic achievement in that region [19~~6~~]. It follows from previous research that parent's socio-economic status, including academic and professional qualifications and income, can be associated with~~impact~~ student success [22~~18~~]. However, in the present study, only the mother's employment status was found to be significantly associated with ~~impact~~ GPA. The university in which students were enrolled was also identified as a factor affecting GPA, possibly attributed to varying resources and facilities offered by each institution. This finding aligns with the significant association observed between students' satisfaction with the quality of education provided by their institution and GPA. Regarding physical and ~~-~~lifestyle factors, a significant association was revealed between smoking and GPA, consistent with lower GPAs among students who smoke, as reported in previous studies [19~~6~~,41~~37~~]. Physical activity also showed an association with GPA, with sedentary students being less likely to achieve high GPAs compared to their active counterparts. This association was previously highlighted in a study among medical students in Saudi Arabia [42~~38~~].

Sleep deprivation is common among university students and has been linked to poor academic performance [43~~39~~]. While a study in Belgium found a positive relationship between sleep quality and academic achievement [20~~17~~], no association was found between sleeping habits or hours and GPA in our study.

4.2. Scientfic and practical recommendations

The results of our study have consequences not just for Lebanon but also for other nearby countries in MENA region, where problems with university students' mental, physical, and academic well-being may be common. The MENA region shares certain issues including economic instability, political turmoil, and the effects of global health crises [44]. Thus, the knowledge gathered from our research could guide the development of interventions and policies meant to promote the academic achievement and general well-being of university students in surrounding countries.

Moreover, our research highlights the necessity of paying more attention to mental health concerns among college students worldwide. The concerning rates of stress, anxiety, and depression that our research revealed are likely not specific to Lebanon; rather, they could be a reflection of larger patterns that influence young adults across a range of cultural contexts [45]. Because of this, international efforts to address mental health on college campuses should be prioritized, with an emphasis on developing supportive learning environments, facilitating access to mental health services.

Formatted: MDPI_2.2_heading2

4.3. Research limitations

Formatted: MDPI_2.2_heading2

The findings of the current study should be interpreted considering the study's limitations. First, our study was cross-sectional, causal relationships cannot be drawn. For instance, students from low-income families may face unique challenges such as limited access to opportunities for physical activities, which could affect~~impact~~ their academic performance. Moreover, time constraints may be associated with~~impact~~ students' ability to dedicate sufficient time to learning. Therefore, future studies using longitudinal designs could be developed to better understand causal relationships. Second, the data collection process utilized self-reported responses to assess the relationship~~impact~~ between~~of~~ both physical and mental health factors with~~on~~ the academic success of university students. However, it's important to acknowledge the potential for inaccuracies stemming from memory recall or social desirability bias within these responses. Third, this study primarily employed quantitative methods for assessment. Future research endeavors may benefit from incorporating qualitative approaches to further explore the relationship~~ impact~~ between~~of~~ physical health, ~~and~~ mental health and~~on~~ university students' success, thereby offering a more comprehensive understanding of the subject matter.

5. Conclusions

The present study shed light on the alarming levels of depression, anxiety, and stress reported among college students. Our findings highlighted the association between mental and physical health with GPA, emphasizing the critical need for targeted interventions to support student well-being and academic success. Policymakers and universities must develop and implement awareness campaigns and health education initiatives tailored specifically to the needs of university students. In order to reduce the negative impact of mental health issues on academic performance, these programs should emphasize the promotion of a healthy lifestyle, which includes regular physical activity, a balanced diet, and enough sleep. Furthermore, it is critical to create a welcoming campus environment that promotes open dialogue about mental health and easily accessible options for students in need of support.

Author Contributions: Conceptualization, SAK, NA, SJC, TA, RB; methodology, SAK; software, SAK and RB; validation, SAK, NA, SJC, TA, RB; formal analysis, SAK and RB; investigation, SAK and RB; resources, SAK, NA, SJC, TA, RB; data curation, SAK and RB; writing—original draft preparation, SAK, NA, SJC, TA, RB; writing—review and editing, SAK, NA, SJC, TA, RB; visualization, SAK and RB; supervision, SAK; project administration, SAK, NA; funding acquisition, SAK. All authors have read and agreed to the published version of the manuscript.

Funding: This research was funded by the University Research Board and the Board Designated Professorship at the American University of Beirut, Lebanon.

Institutional Review Board Statement: The study was conducted in accordance with the Declaration of Helsinki and approved by the Institutional Review Board (or Ethics Committee) of the American University of Beirut (protocol code SBS-2023-0247 and date of approval 28-9-2023).

Informed Consent Statement: Informed consent was obtained from all subjects involved in the study.

Data Availability Statement: The data presented in this study are available on request from the corresponding author. The data are not publicly available due to privacy of participants and ethical concerns.

Acknowledgments: SAK would like to thank the University Research Board and the Board Designated Professorship at the American University of Beirut for funding this study.

Conflicts of Interest: The authors declare no conflicts of interest.

References

1. Spiel, C., Schwartzman, S., Busemeyer, M. R., Cloete, N., Drori, G. S., Lassnigg, L., Schober, B., Schweisfurth, M., Verma, S., Bakarat, B., Maassen, P., & Reich, R. (2018). The contribution of education to social progress*. In Cambridge University Press eBooks (pp. 753–778). https://doi.org/10.1017/9781108399661.006
2. Jayanthi, S. V., Balakrishnan, S., Ching, A. L. S., Latiff, N. a. A., & Nasirudeen, A. (2014). Factors contributing to academic performance of students in a tertiary institution in Singapore. American Journal of Educational Research, 2(9), 752–758. https://doi.org/10.12691/education-2-9-8
3. Altman, R. L., & Wilson, J. H. (2017). Predictors of academic achievement as measured by GPA- PILOT DATA. ResearchGate. https://www.researchgate.net/publication/314538481_Predictors_of_Academic_Achievement_as_Measured_by_GPA-_PILOT_DATA
4. Howard, AL, Carnrite, KD & Barker, ET (2021) First-year university students' mental health trajectories were disrupted at the onset of COVID-19, but disruptions were not linked to housing and financial vulnerabilities: a registered report. Emerg Adulthood 10(1), https://doi.org/10.1177/21676968211053523
5. Campbell, F., Blank, L., Cantrell, A., Baxter, S., Blackmore, C., Dixon, J., & Goyder, E. (2022). Factors that influence mental health of university and college students in the UK: a systematic review. BMC Public Health, 22(1). https://doi.org/10.1186/s12889-022-13943-x
6. Klein, A. M., Wolters, N., Bol, E., Koelen, J., De Koning, L., Roetink, S., Blom, J., Pronk, T., Van Der Heijde, C., Salemink, E., Bolinski, F., Riper, H., Karyotaki, E., Cuijpers, P., Schneider, S., Rapee, R. M., & Vonk, P. (2021). Online computer or therapist-guided cognitive behavioral therapy in university students with anxiety and/or depression: study protocol of a randomised controlled trial. BMJ Open, 11(11), e049554. https://doi.org/10.1136/bmjopen-2021-049554
7. Greenberg, P. B., Kessler, R. C., Birnbaum, H. G., Leong, S. A., Lowe, S. R., Berglund, P. A., & Corey-Lisle, P. K. (2003). The Economic Burden of Depression in the United States. The Journal of Clinical Psychiatry, 64(12), 1465–1475. https://doi.org/10.4088/jcp.v64n1211
8. Roberts, R., Golding, J. B., Towell, T., Reid, S., Woodford, S., Vetere, A., & Weinreb, I. (2000). Mental and physical health in students: The role of economic circumstances. British Journal of Health Psychology, 5(3), 289–297. https://doi.org/10.1348/135910700168928
9. Kasparek, D. G., Corwin, S. J., Valois, R. F., Sargent, R. G., & Morris, R. W. (2008). Selected Health Behaviors That Influence College Freshman Weight Change. Journal of American College Health, 56(4), 437–444. https://doi.org/10.3200/jach.56.44.437-444
10. Sogari, G., Velez-Argumedo, C., Gómez, M. I., & Mora, C. (2018). College Students and Eating Habits: A study using an Ecological Model for Healthy behavior. Nutrients, 10(12), 1823. https://doi.org/10.3390/nu10121823
11. Vainshelboim, B., Bopp, C. M., Wilson, O. W. A., Papalia, Z., & Bopp, M. (2019). Behavioral and Physiological Health-Related Risk Factors in College students. American Journal of Lifestyle Medicine, 15(3), 322–329. https://doi.org/10.1177/1559827619872436
12. Haddad, S. E. (2002). Cultural diversity and sectarian attitudes in postwar Lebanon. Journal of Ethnic and Migration Studies, 28(2), 291–306. https://doi.org/10.1080/13691830220124341
13. Maalouf, F. T., Ghandour, L., Halabi, F., Zeinoun, P., Shehab, A. a. S., & Tavitian, L. (2016). Psychiatric disorders among adolescents from Lebanon: prevalence, correlates, and treatment gap. Social Psychiatry and Psychiatric Epidemiology, 51(8), 1105–1116. https://doi.org/10.1007/s00127-016-1241-4
14. Halat, D. H., Younes, S., Safwan, J., Akiki, Z., Akel, M., & Rahal, M. (2022). Pharmacy Students' Mental Health and Resilience in COVID-19: An Assessment after One Year of Online Education. European Journal of Investigation in Health, Psychology and Education, 12(8), 1082–1107. https://doi.org/10.3390/ejihpe12080077
15. Suzanne, A. A. (2020, November 4). The deteriorated educational reality in Lebanon: Towards "Another" Critical approach. Arab Reform Initiative. https://www.arab-reform.net/publication/the-deteriorated-educational-reality-in-lebanon-towards-another-critical-approach/
16. Itani, R., Mattar, L., Kharroubi, S. A., Bosqui, T., Diab-El-Harake, M., & Jomaa, L. (2022). Food insecurity and mental health of college students in Lebanon: a cross-sectional study. Journal of Nutritional Science, 11. https://doi.org/10.1017/jns.2022.68
17. Fawaz, M., & Samaha, A. A. (2020). E-learning: Depression, anxiety, and stress symptomatology among Lebanese university students during COVID-19 quarantine. Nursing Forum, 56(1), 52–57. https://doi.org/10.1111/nuf.12521
18. Fadel, S., Fahda, S., Akel, M., Rahal, M., Malhab, S. B., Haddad, C., & Dimassi, A. (2023). Mental health assessment of Lebanese pharmacy students after returning to school post-COVID-19: A cross-sectional study. Pharmacy Education, 23(1), 180–192. https://doi.org/10.46542/pe.2023.231.180192
19. Halat, D. H., Hallit, S., Younes, S., AlFikany, M., Khaled, S., Krayem, M., Khatib, S. E., & Rahal, M. (2023). Exploring the effects of health behaviors and mental health on students' academic achievement: a cross-sectional study on lebanese university students. BMC Public Health, 23(1). https://doi.org/10.1186/s12889-023-16184-8

Formatted: No underline, Font color: Black

Formatted: No underline, Font color: Black

20. Baert, S., Omey, E., Verhaest, D., & Vermeir, A. (2015). Mister Sandman, bring me good marks! On the relationship between sleep quality and academic achievement. Social Science & Medicine, 130, 91–98. https://doi.org/10.1016/j.socscimed.2015.02.011
21. Cambridge University Reporter, 26 February 2003. (n.d.). https://www.admin.cam.ac.uk/reporter/2002-03/weekly/5915/
22. Ali, S., Haider, S. Z., Munir, F., Khan, H., & Ahmed, A. M. (2013). Factors contributing to the students' academic performance: A case study of Islamia University Sub-Campus. American Journal of Educational Research, 1(8), 283–289. https://doi.org/10.12691/education-1-8-3
23. Kroenke, K., & Spitzer, R. L. (2002). The PHQ-9: A new Depression Diagnostic and Severity Measure. Psychiatric Annals, 32(9), 509–515. https://doi.org/10.3928/0048-5713-20020901-06
24. Sawaya, H., Atoui, M., Hamadeh, A., Zeinoun, P., & Nahas, Z. (2016). Adaptation and initial validation of the Patient Health Questionnaire – 9 (PHQ-9) and the Generalized Anxiety Disorder – 7 Questionnaire (GAD-7) in an Arabic speaking Lebanese psychiatric outpatient sample. Psychiatry Research, 239, 245–252. https://doi.org/10.1016/j.psychres.2016.03.030
25. Malik, M. N., & Javed, S. (2021). Perceived stress among university students in Oman during COVID-19-induced e-learning. Middle East Current Psychiatry, 28(1). https://doi.org/10.1186/s43045-021-00131-7
26. Naal, H., Tavitian-Elmadjian, L., & Yacoubian, H. A. (2020). Predictors of mental health literacy in a sample of university students in Lebanon. International Journal of Mental Health, 51(4), 381–403. https://doi.org/10.1080/00207411.2020.1838239
27. Kronfol, Z., Khalifa, B., Khoury, B., Omar, O., Daouk, S., DeWitt, J., ElAzab, N., & Eisenberg, D. (2018). Selected psychiatric problems among college students in two Arab countries: comparison with the USA. BMC Psychiatry, 18(1). https://doi.org/10.1186/s12888-018-1718-7
28. Meckamalil, C., Brodie, L., Hogg-Johnson, S., Carroll, L., Jacobs, C., & Côté, P. (2020). The prevalence of anxiety, stress and depressive symptoms in undergraduate students at the Canadian Memorial Chiropractic College. Journal of American College Health, 70(2), 371–376. https://doi.org/10.1080/07448481.2020.1751173
29. Flaherty, C. (2023). How college students rate campus health and wellness offerings. Inside Higher Ed | Higher Education News, Events and Jobs. https://www.insidehighered.com/news/student-success/health-wellness/2023/05/31/how-college-students-rate-campus-health-and
30. Eisenberg, D., Hunt, J., & Speer, N. K. (2013). Mental Health in American Colleges and Universities. Journal of Nervous and Mental Disease, 201(1), 60–67. https://doi.org/10.1097/nmd.0b013e31827ab077
31. Ansari, W. E., Labeeb, S. A., Moseley, L., Kotb, S. A., & El-Houfy, A. A. (2013). Physical and Psychological Well-being of University Students: Survey of Eleven Faculties in Egypt. PubMed. https://pubmed.ncbi.nlm.nih.gov/23626886
32. Peltzer, K., Pengpid, S., Samuels, T. A., Özcan, N. K., Mantilla, C., Rahamefy, O. H., Wong, M. L., & Gasparishvili, A. (2014). Prevalence of Overweight/Obesity and Its Associated Factors among University Students from 22 Countries. International Journal of Environmental Research and Public Health, 11(7), 7425–7441. https://doi.org/10.3390/ijerph110707425
33. Yahia, N., Achkar, A., Abdallah, A., & Rizk, S. (2008). Eating habits and obesity among Lebanese university students. Nutrition Journal, 7(1). https://doi.org/10.1186/1475-2891-7-32
34. Boukrim, M., Obtel, M., Lahlou, L., & Razine, R. (2021). University students' perceptions and factors contributing to obesity and overweigh in Southern of Morocco. African Health Sciences, 21(2), 942–950. https://doi.org/10.4314/ahs.v21i2.56
35. Makkawy, E., Alrakha, A. M., Almubarak, A. F., Alotaibi, H. T., Alotaibi, N. T., Alasmari, A. A., & Altamimi, T. (2021). Prevalence of overweight and obesity and their associated factors among health sciences college students, Saudi Arabia. Journal of Family Medicine and Primary Care, 10(2), 961. https://doi.org/10.4103/jfmpc.jfmpc_1749_20
36. He, J., Chen, X., Fan, X., Cai, Z., & Huang, F. (2019). Is there a relationship between body mass index and academic achievement? A meta-analysis. Public Health, 167, 111–124. https://doi.org/10.1016/j.puhe.2018.11.002
37. Finn, K. E., Faith, M. S., & Seo, Y. S. (2018). School engagement in relation to body mass index and school achievement in a High-School Age sample. Journal of Obesity, 2018, 1–7. https://doi.org/10.1155/2018/3729318
38. Alfifi, H. Y., & Abed, J. (2017). Factors contributing to students' academic performance in the Education College at Dammam University. Education Journal, 6(2), 77. https://doi.org/10.11648/j.edu.20170602.11
39. Tadese, M., Yeshaneh, A., & Mulu, G. B. (2022). Determinants of good academic performance among university students in Ethiopia: a cross-sectional study. BMC Medical Education, 22(1). https://doi.org/10.1186/s12909-022-03461-0
40. Mehare, T., Kassa, R., Mekuriaw, B., & Mengesha, T. (2020). Assessing predictors of academic Performance for NMEI Curriculum-Based medical students found in the Southern Ethiopia. Education Research International, 2020, 1–8. https://doi.org/10.1155/2020/8855306
41. Ong, C. K. Y., Hutchesson, M. J., Patterson, A., & Whatnall, M. (2021). Is There an Association between Health Risk Behaviours and Academic Achievement among University Students? International Journal of Environmental Research and Public Health, 18(16), 8314. https://doi.org/10.3390/ijerph18168314
42. AlDrees, A., Abdulghani, H. M., Irshad, M., Baqays, A., Al-Zhrani, A. A., Alshammari, S. A., & Alturki, N. I. (2016). Physical activity and academic achievement among the medical students: A cross-sectional study. Medical Teacher, 38(sup1), S66–S72. https://doi.org/10.3109/0142159x.2016.1142516
43. Patrick, Y., Lee, A. C., Raha, O., Pillai, K., Gupta, S., Sethi, S., Mukeshimana, F., Gerard, L., Moghal, M., Saleh, S. N., Smith, S. M., Morrell, M. J., & Moss, J. N. (2017). Effects of sleep deprivation on cognitive and physical performance in university students. Sleep and Biological Rhythms, 15(3), 217–225. https://doi.org/10.1007/s41105-017-0099-5

Formatted: No underline, Font color: Black

44. Katoue, M. G., Cerda, A. A., García, L. Y., & Jakovljević, M. (2022). Healthcare system development in the Middle East and North Africa region: Challenges, endeavors and prospective opportunities. Frontiers in Public Health, 10. https://doi.org/10.3389/fpubh.2022.1045739
45. Van Der Heijde, C., Vonk, P., & Meijman, F. J. (2015). Self-regulation for the promotion of student health. Traffic lights: the development of a tailored web-based instrument providing immediate personalized feedback. Health Psychology and Behavioral Medicine, 3(1), 169–189. https://doi.org/10.1080/21642850.2015.1049950

1. Spiel, C., Schwartzman, S., Busemeyer, M. R., Cloete, N., Drori, G. S., Lassnigg, L., Schober, B., Schweisfurth, M., Verma, S., Bakarat, B., Maassen, P., & Reich, R. (2018). The contribution of education to social progress*. In Cambridge University Press eBooks (pp. 753–778). https://doi.org/10.1017/9781108399661.006
2. Jayanthi, S. V., Balakrishnan, S., Ching, A. L. S., Latiff, N. a. A., & Nasirudeen, A. (2014). Factors contributing to academic performance of students in a tertiary institution in Singapore. American Journal of Educational Research, 2(9), 752–758. https://doi.org/10.12691/education-2-9-8
3. Cambridge University Reporter, 26 February 2003. (n.d.). https://www.admin.cam.ac.uk/reporter/2002-03/weekly/5915/
4. Howard, AL, Carnrite, KD & Barker, ET (2021) First-year university students' mental health trajectories were disrupted at the onset of COVID-19, but disruptions were not linked to housing and financial vulnerabilities: a registered report. Emerg Adulthood 10(1), https://doi.org/10.1177/21676968211053523
5. Campbell, F., Blank, L., Cantrell, A., Baxter, S., Blackmore, C., Dixon, J., & Goyder, E. (2022). Factors that influence mental health of university and college students in the UK: a systematic review. BMC Public Health, 22(1). https://doi.org/10.1186/s12889-022-13943-x
6. Greenberg, P. B., Kessler, R. C., Birnbaum, H. G., Leong, S. A., Lowe, S. R., Berglund, P. A., & Corey-Lisle, P. K. (2003). The Economic Burden of Depression in the United States. The Journal of Clinical Psychiatry, 64(12), 1465–1475. https://doi.org/10.4088/jcp.v64n1211
7. Roberts, R., Golding, J. B., Towell, T., Reid, S., Woodford, S., Vetere, A., & Weinreb, I. (2000). Mental and physical health in students: The role of economic circumstances. British Journal of Health Psychology, 5(3), 289–297. https://doi.org/10.1348/135910700168928
8. Kasparek, D. G., Corwin, S. J., Valois, R. F., Sargent, R. G., & Morris, R. W. (2008). Selected Health Behaviors That Influence College Freshman Weight Change. Journal of American College Health, 56(4), 437–444. https://doi.org/10.3200/jach.56.44.437-444
9. Maalouf, F. T., Ghandour, L., Halabi, F., Zeinoun, P., Shehab, A. a. S., & Tavitian, L. (2016). Psychiatric disorders among adolescents from Lebanon: prevalence, correlates, and treatment gap. Social Psychiatry and Psychiatric Epidemiology, 51(8), 1105–1116. https://doi.org/10.1007/s00127-016-1241-4
10. Halat, D. H., Younes, S., Safwan, J., Akiki, Z., Akel, M., & Rahal, M. (2022). Pharmacy Students' Mental Health and Resilience in COVID-19: An Assessment after One Year of Online Education. European Journal of Investigation in Health, Psychology and Education, 12(8), 1082–1107. https://doi.org/10.3390/ejihpe12080077
11. Suzanne, A. A. (2020, November 4). The deteriorated educational reality in Lebanon: Towards "Another" Critical approach. Arab Reform Initiative. https://www.arab-reform.net/publication/the-deteriorated-educational-reality-in-lebanon-towards-another-critical-approach/
12. Itani, R., Mattar, L., Kharroubi, S. A., Bosqui, T., Diab-El-Harake, M., & Jomaa, L. (2022). Food insecurity and mental health of college students in Lebanon: a cross-sectional study. Journal of Nutritional Science, 11. https://doi.org/10.1017/jns.2022.68
13. Fawaz, M., & Samaha, A. A. (2020). E-learning: Depression, anxiety, and stress symptomatology among Lebanese university students during COVID-19 quarantine. Nursing Forum, 56(1), 52–57. https://doi.org/10.1111/nuf.12521
14. Fadel, S., Fahda, S., Akel, M., Rahal, M., Malhab, S. B., Haddad, C., & Dimassi, A. (2023). Mental health assessment of Lebanese pharmacy students after returning to school post-COVID-19: A cross-sectional study. Pharmacy Education, 23(1), 180–192. https://doi.org/10.46542/pe.2023.231.180192
15. Haddad, S. E. (2002). Cultural diversity and sectarian attitudes in postwar Lebanon. Journal of Ethnic and Migration Studies, 28(2), 291–306. https://doi.org/10.1080/13691830220124341
16. Halat, D. H., Hallit, S., Younes, S., AlFikany, M., Khaled, S., Krayem, M., Khatib, S. E., & Rahal, M. (2023). Exploring the effects of health behaviors and mental health on students' academic achievement: a cross-sectional study on lebanese university students. BMC Public Health, 23(1). https://doi.org/10.1186/s12889-023-16184-8
17. Baert, S., Omey, E., Verhaest, D., & Vermeir, A. (2015). Mister Sandman, bring me good marks! On the relationship between sleep quality and academic achievement. Social Science & Medicine, 130, 91–98. https://doi.org/10.1016/j.socscimed.2015.02.011
18. Ali, S., Haider, S. Z., Munir, F., Khan, H., & Ahmed, A. M. (2013). Factors contributing to the students' academic performance: A case study of Islamia University Sub-Campus. American Journal of Educational Research, 1(8), 283–289. https://doi.org/10.12691/education-1-8-3
19. Kroenke, K., & Spitzer, R. L. (2002). The PHQ-9: A new Depression Diagnostic and Severity Measure. Psychiatric Annals, 32(9), 509–515. https://doi.org/10.3928/0048-5713-20020901-06

Formatted: Indent: Before: 0.3", No bullets or numbering

20. Sawaya, H., Atoui, M., Hamadeh, A., Zeinoun, P., & Nahas, Z. (2016). Adaptation and initial validation of the Patient Health Questionnaire – 9 (PHQ-9) and the Generalized Anxiety Disorder – 7 Questionnaire (GAD-7) in an Arabic speaking Lebanese psychiatric outpatient sample. Psychiatry Research, 239, 245–252. https://doi.org/10.1016/j.psychres.2016.03.030
21. Malik, M. N., & Javed, S. (2021). Perceived stress among university students in Oman during COVID-19-induced e-learning. Middle East Current Psychiatry, 28(1). https://doi.org/10.1186/s43045-021-00131-7
22. Naal, H., Tavitian-Elmadjian, L., & Yacoubian, H. A. (2020). Predictors of mental health literacy in a sample of university students in Lebanon. International Journal of Mental Health, 51(4), 381–403. https://doi.org/10.1080/00207411.2020.1838239
23. Kronfol, Z., Khalifa, B., Khoury, B., Omar, O., Daouk, S., DeWitt, J., ElAzab, N., & Eisenberg, D. (2018). Selected psychiatric problems among college students in two Arab countries: comparison with the USA. BMC Psychiatry, 18(1). https://doi.org/10.1186/s12888-018-1718-7
24. Meckamalil, C., Brodie, L., Hogg-Johnson, S., Carroll, L., Jacobs, C., & Côté, P. (2020). The prevalence of anxiety, stress and depressive symptoms in undergraduate students at the Canadian Memorial Chiropractic College. Journal of American College Health, 70(2), 371–376. https://doi.org/10.1080/07448481.2020.1751173
25. Flaherty, C. (2023). How college students rate campus health and wellness offerings. Inside Higher Ed | Higher Education News, Events and Jobs. https://www.insidehighered.com/news/student-success/health-wellness/2023/05/31/how-college-students-rate-campus-health-and
26. Eisenberg, D., Hunt, J., & Speer, N. K. (2013). Mental Health in American Colleges and Universities. Journal of Nervous and Mental Disease, 201(1), 60–67. https://doi.org/10.1097/nmd.0b013e31827ab077
27. Ansari, W. E., Labeeb, S. A., Moseley, L., Kotb, S. A., & El-Houfy, A. A. (2013). Physical and Psychological Well-being of University Students: Survey of Eleven Faculties in Egypt. PubMed. https://pubmed.ncbi.nlm.nih.gov/23626886
28. Peltzer, K., Pengpid, S., Samuels, T. A., Özcan, N. K., Mantilla, C., Rahamefy, O. H., Wong, M. L., & Gasparishvili, A. (2014). Prevalence of Overweight/Obesity and Its Associated Factors among University Students from 22 Countries. International Journal of Environmental Research and Public Health, 11(7), 7425–7441. https://doi.org/10.3390/ijerph110707425
29. Yahia, N., Achkar, A., Abdallah, A., & Rizk, S. (2008). Eating habits and obesity among Lebanese university students. Nutrition Journal, 7(1). https://doi.org/10.1186/1475-2891-7-32
30. Boukrim, M., Obtel, M., Lahlou, L., & Razine, R. (2021). University students' perceptions and factors contributing to obesity and overweigh in Southern of Morocco. African Health Sciences, 21(2), 942–950. https://doi.org/10.4314/ahs.v21i2.56
31. Makkawy, E., Alrakha, A. M., Almubarak, A. F., Alotaibi, H. T., Alotaibi, N. T., Alasmari, A. A., & Altamimi, T. (2021). Prevalence of overweight and obesity and their associated factors among health sciences college students, Saudi Arabia. Journal of Family Medicine and Primary Care, 10(2), 961. https://doi.org/10.4103/jfmpc.jfmpc_1749_20
32. He, J., Chen, X., Fan, X., Cai, Z., & Huang, F. (2019). Is there a relationship between body mass index and academic achievement? A meta-analysis. Public Health, 167, 111–124. https://doi.org/10.1016/j.puhe.2018.11.002
33. Finn, K. E., Faith, M. S., & Seo, Y. S. (2018). School engagement in relation to body mass index and school achievement in a High-School Age sample. Journal of Obesity, 2018, 1–7. https://doi.org/10.1155/2018/3729318
34. Alfifi, H. Y., & Abed, J. (2017). Factors contributing to students' academic performance in the Education College at Dammam University. Education Journal, 6(2), 77. https://doi.org/10.11648/j.edu.20170602.11
35. Tadese, M., Yeshaneh, A., & Mulu, G. B. (2022). Determinants of good academic performance among university students in Ethiopia: a cross-sectional study. BMC Medical Education, 22(1). https://doi.org/10.1186/s12909-022-03461-0
36. Mehare, T., Kassa, R., Mekuriaw, B., & Mengesha, T. (2020). Assessing predictors of academic Performance for NMEI Curriculum-Based medical students found in the Southern Ethiopia. Education Research International, 2020, 1–8. https://doi.org/10.1155/2020/8855306
37. Ong, C. K. Y., Hutchesson, M. J., Patterson, A., & Whatnall, M. (2021). Is There an Association between Health Risk Behaviours and Academic Achievement among University Students? International Journal of Environmental Research and Public Health, 18(16), 8314. https://doi.org/10.3390/ijerph18168314
38. AlDrees, A., Abdulghani, H. M., Irshad, M., Baqays, A., Al-Zhrani, A. A., Alshammari, S. A., & Alturki, N. I. (2016). Physical activity and academic achievement among the medical students: A cross-sectional study. Medical Teacher, 38(sup1), S66–S72. https://doi.org/10.3109/0142159x.2016.1142516
39. Patrick, Y., Lee, A. C., Raha, O., Pillai, K., Gupta, S., Sethi, S., Mukeshimana, F., Gerard, L., Moghal, M., Saleh, S. N., Smith, S. M., Morrell, M. J., & Moss, J. N. (2017). Effects of sleep deprivation on cognitive and physical performance in university students. Sleep and Biological Rhythms, 15(3), 217–225. https://doi.org/10.1007/s41105-017-0099-5
